# On the cognitive mechanisms supporting prosocial disobedience in a post-genocidal context

**DOI:** 10.1038/s41598-022-26460-z

**Published:** 2022-12-19

**Authors:** Emilie A. Caspar, Darius Gishoma, Pedro Alexandre Magalhaes de Saldanha da Gama

**Affiliations:** 1 The Moral and Social Brain Lab, Department of Experimental Psychology, Ghent University, Henri Dunantlaan, 2, 9000 Ghent, Belgium; 2 Center for Research in Cognition and Neurosciences, ULB Neuroscience Institute, Université Libre de Bruxelles, Brussels, Belgium; 3 Mental Health Department, College of Medicine and Health Sciences, University of Rwanda, Kigali, Rwanda

**Keywords:** Social behaviour, Social neuroscience

## Abstract

The aim of the present study was to offer a first investigation of the neuro-cognitive processes and the temporal dynamics at the neural level, together with cultural, social and psychological dimensions, that may support resistance to orders to harm another person. Using a novel experimental approach to study experimentally disobedience, we recruited individuals from the first generation born after the 1994 genocide in Rwanda. Seventy-two were recruited and tested in Rwanda and 72 were recruited and tested in Belgium. Results indicated that a higher neural response to the pain of others and a higher feeling of responsibility when people obeyed orders were associated with more resistance to immoral orders. We also observed that participants who had a higher processing, as measured through mid-frontal theta activity, when listening to the orders of the experimenter disobeyed less frequently to immoral orders. Further, participants experiencing a higher conflict before administering a shock to the ‘victim’ also disobeyed more frequently to immoral orders. Finally, a low cultural relationship to authority and a high estimated family suffering during the genocide were also associated with more disobedience to immoral orders. The present study opens new paths for interdisciplinary field research dedicated to the study of obedience.

## Introduction

The history of nations is plagued by horrendous acts of obedience that have caused wars and the loss of countless lives^1^. Such examples have not only been observed in military personnel mandated during wars. Civilians are also capable of atrocious acts to follow authoritative figures^2^. As an example, the Genocide Against Tutsis has been characterized by a high number of civilians who joined the *Interahamwe* militia, the Hutu militia told to be responsible for the 1994 genocide. There are fortunately several examples in history that showed that some individuals risked their own life by resisting orders to save other human beings^3^. However, why some individuals resist immoral orders while others obey such orders remains largely unknown.

Understanding why some people refuse immoral orders while others do not involves answering two main axes of research: (1) which social and situational factors support resistance to immoral orders, and (2) which individual traits and neuro-cognitive differences support resistance to immoral orders. While the first axis has been significantly covered by past experimental research, notably Milgram’s studies^4^ and its subsequent variants^5,6^, the second axis has been much less investigated. A few studies showed that some personality traits may support disobedience (e.g. empathic concern, risk-taking)^7,8^. But studying personality traits supporting disobedience is not sufficient to understand why, in a given situation, some people will refuse orders to harm another person while others will comply with such orders. In the present study, our aim was to offer a first investigation of the neuro-cognitive processes and the temporal dynamics at play when people choose to obey or disobey immoral orders, together with cultural, social and psychological dimensions, which may support resistance to immoral orders. There is a substantial debate around the definition of morality^9^. In the present research, immoral orders refer to orders to inflict physical pain to another person.

When someone obeys or disobeys an order, several cognitive processes are involved at different moments. First, one hears and processes the auditory instruction received from the person giving orders. Then, one decides to obey or not this order, and then executes the corresponding action. Finally, one processes the consequences of their action on others. Translated into neuro-cognitive terms, an investigation of the temporal dynamic of (dis)obedience would involve five key processes, that are: (1) the neural processing of the auditory information when individuals receive the order, (2) the degree of cognitive conflict before acting, (3) the sense of agency over the action and its consequence, (4) the feeling of responsibility over the decision, and (5) empathy for what the victim may feel. The present study aims to investigate the involvement of each of these neuro-cognitive processes to understand how they relate to prosocial disobedience. To do so, behavioural and electrophysiological measurements will be used. Electroencephalography (EEG) allows to study the temporal course of cognition considering its acute temporal resolution.

In the literature, past studies showed that a reduced activity in the frontal midline theta rhythm (FMθ, 4–8 Hz) was associated with reduced conscious attention of the auditory processing^10^ and reduced vigilance to auditory stimuli^11^. In the present study, we investigated to what extent the processing of the auditory instructions to physically harm another individual or not influenced the extent to which participants disobeyed orders. To the best of our knowledge, no previous studies investigated to what extent the processing of auditory information influences subsequent obedience behaviors. However, past theoretical work suggested to the more people would distance themselves from the experimenter, the less they would comply with orders^12^. We thus expected that individuals who distance themselves from the orders of the experimenter and disengage their attention from the orders received, as reflected by a lower FMθ, would disobey more frequently.

Cognitive conflict occurs when two competing response options compete for control of behavior^13^. Previous studies indicated that a higher cognitive conflict involves that the selected action was not the most natural to select compared to the other actions, which can lead to a change in behaviors^14^. Using EEG, past literature has shown that a stronger conflict elicits a higher activity in the FMθ compared to low conflicts^15–19^. Here, we expected that those having a high FMθ, indicating a high cognitive conflict, before obeying the orders to harm another person would refuse more frequently those orders.

The sense of agency has been mainly described as the feeling that we are the authors of, and thus potentially responsible for, our own actions and their consequences in the external world^20^. In the literature, the sense of agency has been measured with explicit methods, but those methods can be biased by social desirability^21^. An alternative, implicit measure of the sense of agency based on time estimation of action-outcome intervals has thus been used^22^. In classic time estimation tasks, participants have to estimate the duration of a time interval that elapsed between an action (e.g., pressing a button) and its consequences (e.g., hearing the beep it produces). Results typically show that participants tend to estimate time intervals as shorter when the action is performed voluntarily compared to a condition in which the action is performed involuntarily^23^, an effect referred to as temporal binding. The feeling of responsibility is related, but refers to a mental representation of authorship in a social context^24^. It has been mostly measured with explicit questions asked to the participants^25,26^. Previous work showed that when individuals obey orders, their sense of agency and their feeling of responsibility are attenuated compared to a situation where they are free to decide which action to execute^27–29^. Another study indicated that temporoparietal junction (TPJ) subserves the sense of agency^30^. The authors showed that cathodal stimulation over the right temporoparietal junction (rTPJ) reduced reaction times to harm another person in a situation of obedience. Finally, another recent study showed that a reduced sense of agency, as measured with the method of interval estimates, and a low feeling of responsibility correlated with a high number of shocks sent to a victim^31^. We thus predicted that a higher sense of agency and a higher feeling of responsibility, would be correlated with a higher prosocial disobedience.

Empathy for pain has been defined as our capacity to feel others' pain, including the pain that we can cause them^32^. Empathy can be measured through several measurements, from subjective reports to indirect neuroimaging measurements. An extensive literature using neuroimaging has shown that seeing another individual in pain triggers an empathic response in the brain of the observer, especially in the insula and in the anterior cingulate cortex. In EEG studies, a recent meta-analysis suggested that the P3 and the Late Positive Potential (LPP) are robust to measure the neural response to the pain of others^33^. Previous research showed that a higher neural response to the pain of others is linked to a higher prosociality^31,34,35^. Refusing to obey an immoral order is also a prosocial behaviour and could thus rely on similar mechanisms. We thus expected a higher amplitude of the P3 and the LPP to be associated with a higher prosocial disobedience.

Past scientific literature on (dis)obedience is most exclusively based on Milgram’s study^36^ or its subsequent variants, which demonstrated a strong willingness to follow the orders of an experimenter. However, Milgram’s studies posed serious ethical issues, such as the use of deception, the high stress caused to the participants and the fact that participants were in the role of the perpetrator only^37^. Further, the subsequent variants of Milgram’s studies^5,6^ were not adapted for neuro-cognitive measurements. Recently, a novel experimental paradigm has been developed to study disobedience^38^. In this paradigm, two volunteers are recruited and take turns to be ‘agents’ or ‘victims’, making the procedure fully reciprocal and avoiding having people in the role of the perpetrator only. For each trial, the agents receive an order from the experimenter to send a real, mildly painful electric shock to the ‘victim’, thus placing participants in an ecological set-up and avoiding the use of cover stories and deception. These shocks lead to either a small monetary gain or no monetary gain at all. In this paradigm, disobedience to immoral orders (i.e., prosocial disobedience) corresponds to the number of times agents refused the orders of the experimenter to send a painful shock to the ‘victim’. Several variants have been tested on university students in Belgium and results indicated that the prosocial disobedience rate ranged between 7.03 and 60.88%, thus indicating that it can be used to study disobedience. Importantly, stress is very minimal with this novel experimental approach^38^. As this paradigm is adapted to neuro-cognitive measurements, we used it in the present study. We tested three variants of the same task to create variability in prosocial disobedience to subsequently perform correlations. In previous work^38^, receiving money for each shock was a main factor modulating prosocial disobedience by about 20%. In one variant of the present task, participants thus received money for each shock sent, and in another variant, they did not receive a monetary reward. In a third variant, we explicitly told participants that they were free to decide to send shocks or not in exchange for money despite the orders of the experimenter.

Critically, the classical WEIRD (western, educated, industrialized, rich & democratic) population used in neuroscience is not frequently facing extreme situations of obedience and their dramatic consequences. In the present study, we thus decided to test our predictions on the new generation of Rwandese born after the genocide instead of more classical western university students because growing up amidst or in the aftermath of wars has strong effects on individuals^39^. In Rwanda, the new generation has grown up in a context in which obedience to authority is a huge concern. Genocides are indeed frequently referred to as ‘crimes of obedience’^40^, with a majority of perpetrators complying with orders to exterminate another group as the commonality. In Rwanda, the ‘following bad leaders’ statement is a popular explanation for the genocide^41^, emphasizing specific concern regarding the consequences of blind obedience.

We decided to explore if Rwandese leaving in Rwanda or Rwandese leaving in a different country would have similar results regarding prosocial disobedience and the related processes. Migrating to another country lead to cultural and psychological changes resulting from the direct contact with people from another culture^42–44^. This phenomenon, referred to as acculturation^43^, has also been shown to occur in migrants’ children^45^. In the present study, to enlarge our understanding of the cultural and psychological factors influencing prosocial disobedience, we thus compared two groups of participants: a group composed of Rwandese born after the genocide and currently living in Rwanda and a group composed of Rwandese born after the genocide and living in Belgium. However, to the best of our knowledge no previous studies compared the same constructs on these two groups of individuals. Our comparisons between the two groups on the relationship to money, relationship to authority, on moral foundations and to the identification to the experimenter were thus exploratory and not hypothesis driven.

As people’s decision to comply with immoral orders can be influenced by several cultural, social and psychological factors^46,3^, we also integrated several additional predictor variables for prosocial disobedience. Deference to authority has been emphasized as a factor of importance to explain the genocide in Rwanda^47,48^ and was thus added as a predictor variable with the aggression-submission-conventionalism scale^49^. Humans are sensitive to different competing issues of morality, which has been identified as a key reason for rescuing persecuted people^50^. We thus also integrated the moral foundation questionnaire^51^. Since participants could receive a small monetary reward for each shock, we also evaluated their attitude to money with the money attitude scale^52^. It had also been argued that identification with the authority promotes obedience^53^. We thus also integrated a questionnaire on the identification with the experimenter^12^.

Past work suggested that historical victimization encourages a sense of moral obligation to reduce the suffering of others^54^, thus implying an increased prosociality. However, previous research largely ignored individual-level behaviors, especially in the context of obedience to authority, focusing mostly on the population level. Further, no previous studies sought to understand the psychological and neural mechanisms influencing individual behaviors in this context. In the present study, we thus also examined to what extent our participants estimated that their family suffered during the genocide, and to what extent they participate in reconciliation activities relates to prosocial disobedience.

## Method and material

### Participants

One hundred forty-four Rwandese were recruited for this experiment. Seventy-two were recruited and tested in Rwanda (4 women, 68 men) and 72 (42 women, 30 men) were recruited and tested in Belgium. A priori computation of sample size indicates that we needed to recruit 134 participants (= 67 pairs) to detect a correlation (two-tailed) of 0.30 with 95% power (Cohen’s convention)^55^. We decided to increase this number up to 144 (= 72 pairs) in order to prevent potential loss of data. The discrepancy between the number of male and female participants in the group tested in Rwanda was due to the current female hairstyle in Rwanda (i.e., braided hairs in a ponytail), which is not compatible with electroencephalography (EEG) recordings. We recruited participants studying at university to facilitate comprehension since the official language in universities in Rwanda is English, but also to control for the educational level, as participants tested in Belgium were also university students. In both countries, participants had to have at least one parent from Rwanda. We selected participants between 18 and 26 years old in order to have a sample composed of individuals who were not born yet when the genocide happened in 1994. The mean age was 23.36 (SD = 1.60) for the group of Rwandese tested in Rwanda and 22.11 (SD = 2.65) for the group of Rwandese tested in Belgium. The following exclusion criteria were determined prior to further analysis: (1) failure to understand the task, (2) failure to perform the interval estimate task used as a proxy for sense of agency correctly, or (3) failure to obtain good signal-to-noise ratio for EEG recordings. Classic reasons to fail to obtain a good signal-to-noise ratio involve eye movement or head movement artefacts, or no clear Event-Related Potentials (ERPs). For the group tested in Rwanda, we had to increase those reasons to the following: 50 Hz noise (i.e., power noise) because there was no ground plug in the room we were given to conduct the experiment; heavy rain or numerous crows on the sheet metal roof that did not allow the participants to hear auditory stimuli; power cuts during the testing; or sweat artefacts due to hot temperatures. For the group tested in Belgium, the full data of one participant were not acquired due to a late arrival and the lack of time to finish the role reversal procedure. In the group tested in Rwanda, we lost the EEG data of 3 participants, 1 because of the high number of eye blinks, 1 because of noisy crows on the metal sheet roof and 1 because of the noise of the rain. In the group tested in Belgium, we lost the EEG data of 4 participants, 1 participant because of visual artefacts, 1 because of sweat artefacts and 2 because of a problem with CMS/DRL electrodes. Of note, for some participants we could only record central electrodes (i.e., Fz, Cz, and Pz) because their (braided) haircut did not allow to obtain a reliable signal on other electrodes. Other data were not removed for those participants. To identify participants for whom the action-tone intervals did not gradually increase with action-tone intervals, we performed a linear trend analysis with contrast coefficients − 1, 0, 1 for the three delays we used, similarly to previous studies^27^. The data of 40/72 participants in the group tested in Rwanda were excluded due to a non-significant linear trend analysis. For the group tested in Belgium, the data of 38/72 participants were lost due to a non-significant linear trend analysis. Of note, this amount of non-reliable data for this task was not predicted and is thus further discussed in the general discussion. Again, other data were not removed for those participants in order to keep a reliable sample size for all the other measurements. The study and its prior hypotheses were approved by the National Ethics Committee of Rwanda (permission 710/RNEC/2019) for the group tested in Rwanda and by the local ethical committee of the Université libre de Bruxelles and the Ethics Committee Erasme Hospital (permission 053/2019) for the group tested in Belgium. For this study all methods were carried out in accordance with relevant guidelines and regulations. Informed consent was obtained from all participants with a method approved by the two ethics committees, ensuring that any potential cultural differences for giving a consent would be considered.

### Material and procedure

Participants were invited by same-gender pairs to participate in this study. We ensured that they were not close friends or relatives. Upon arrival in the laboratory, participants were invited to sit, and they were explained the task in detail, both in English and Kinyarwanda for the group tested in Rwanda and in French for the group tested in Belgium. Both participants filled in the written consent in front of each other and were reminded that they could stop the study at any time.

Participants were explained that there were two roles in this experiment, one of ‘agent’ and one of ‘victim’. They were randomly assigned to start in one of the two roles, but their roles were switched at the middle of the experiment. Thus, some participants first performed the task as agent and then became victim, while other participants had the opposite role reversal procedure. They were told that agents would receive instructions from the main experimenter to either give a painful shock or not to the ‘victim’. They were then told that they will have two buttons: a SHOCK button and a NO SHOCK button. Participants were told the exact following wording: “*I will give you instructions to give a shock or not to the victim and you will have a keyboard with two buttons, one labelled ‘shock’ and one labelled ‘no shock’. You will have to press one of the two buttons after receiving my instructions*”. There were 3 variants of the same task (N = 48 per variant, with N = 24 tested in both countries). In the ‘Do not decide + monetary gain’ variant, if they pressed the SHOCK button, they delivered a painful electric shock to the ‘victim’ in exchange for 50RWF (in Rwanda) or €0.05 (in Belgium). If they pressed the NO SHOCK button, they did not deliver the painful shock to the ‘victim’ and they did not earn additional money. In the ‘Do not decide + no monetary gain’, the financial reward was removed for each shock delivered. In the ‘Decide + monetary gain’ variant, participants earned the financial reward for each shock, but they were explicitly told that, as adults, they remained free to decide to send or not the shock to the ‘victim’. Importantly to mention, at no moment participants were explicitly incentivized to obey orders. We neither explicitly mentioned that they had to follow the orders of the experimenter, nor that we needed them to obey for the sake of the experiment. We also did not react when they pressed the ‘wrong’ button (i.e., for instance, if the experimenter said to ‘give a shock’ and the participant pressed the NO SHOCK button). In addition to the consent forms, we also asked them to fill a document that explicitly stated that they understood that they would receive the remuneration for their time for this experiment, no matter their decisions during the task, see Supplementary Material S1. This procedure was decided to ensure that participants understood that they would be paid even if they refused to follow the instructions of the experimenter. Thus, participants were never explicitly incentivized to disobey orders, but they were not incentivized to follow orders neither, such as in Milgram’s studies^6^ in which participants were explicitly solicited to follow orders. We could thus evaluate their spontaneous propensity to disobey orders. In the variant where they could decide, participants also had to fill in a document that explicitly stated that they understand they could disobey orders (see Supplementary Material S1).

**Figure 1:**
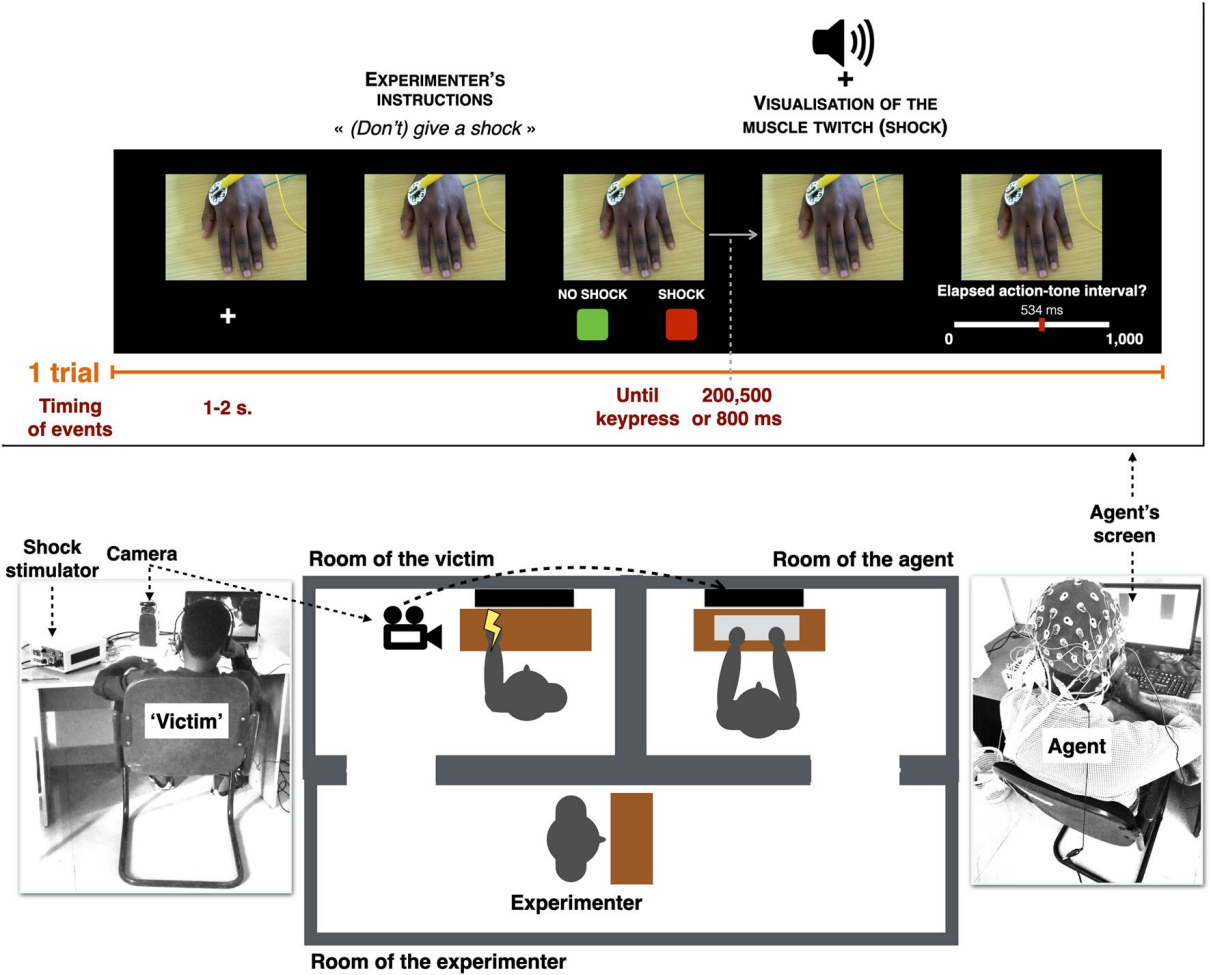
Schematic representation of the experimental set-up. ‘Agents’ and ‘victims’ were located in two separated rooms. On each trial, agents received an order from the experimenter to give or not to give a shock to the ‘victim’ through headphones and had to press either on the SHOCK button or on the NO SHOCK button. Agents could see the consequences of their actions through a real-time video recording displaying the victim’s hand on their own screen.

By picking randomly a card in a box, participants were assigned to start either as agent or victim but were offered the possibility to change if they wanted to. The participant who was in the role of the agent was brought in an isolated room and the ‘victim’ was bought in another room (see Fig. 1).

Two electrodes were placed on the victim’ left hand on the abductor pollicis muscle in order to produce a clear and visible muscle twitch and the threshold was increased by steps of 1 mA until a painful stimulation was achieved following the procedure described in^27^. Participants were told that this selected threshold would neither increase nor decrease during the experiment. The ‘victim’ was asked to place her hand on the table in front of a camera and not to move it during the session. The victim was invited to watch a neutral documentary to make the time pass.

The agent was told that they would be isolated from both the victim and the experimenter in order to avoid visual distractions during EEG recordings. In reality two reasons explained the decision to isolate the agent. First, a previous study reported that when the experimenter is not present in the room, disobedience to immoral orders increases^4^. Agents were given headphones and were told that they would be able to hear the instructions of the experimenter who would be giving orders through a microphone connected to the headphones from another room. Second, since we were recording Event-Related Potentials (ERPs) associated with the visualization of pain on the victim’s hand (i.e., with visible muscular twitch), having a real-time display of the victim’s hand on an adjusted and fixed screen would better prevent head and eye movements.

Each trial started with a fixation cross lasting between 1 and 2 s. When the fixation cross disappeared, the experimenter told the agent either ‘*give a shock*’ or ‘*don’t give a shock*’. In reality, these sentences were pre-recorded. To increase the authenticity of the procedure, each sentence was recorded 6 times with small variations in the voice and displayed randomly. In addition, the audio recordings included a background sound similar to interphone communications. After receiving the verbal order, a picture of two rectangles, a red one labelled ‘SHOCK’ and a green one labelled ‘NO SHOCK’, was displayed on the left and right bottom of the screen. The key-outcome mapping varied randomly on a trial-wise basis, but the outcome was always fully congruent with the mapping seen by the participant. Agents could then press one of the two buttons. A beep followed each keypress by 200, 500 or 800 ms, according to the method action-outcome interval estimates used as a proxy for the sense of agency. If the SHOCK button was pressed, agents witnessed a muscular twitch on the victim’s hand displayed at the same time as the tone. An analogue scale with ‘0’ on the left side of the scale and ‘1000’ on the right side was then displayed on the screen and participants were asked to estimate the duration of the action-tone interval in ms. A red position marker was displayed on that scale with a number, corresponding to the marker’s current position in ms. The starting position of the marker varied randomly on a trial-wise basis and participants were told to ignore the starting position of that scale when providing their final answer. By using the same two buttons as for shock and no shock, participants could move the position of the cursor on that scale to the left or to the right. By pressing for a long time, the cursor moved by steps of ± 50 and by pressing for a short time, the cursor moved by steps of ± 1. After a fixed duration of 6 s, their answer was saved, and the next trial started. Each participant started with a training session to practice the time interval procedure. The training session lasted for a minimum of 8 trials and was repeated until participants declared that they could perform the task correctly. There was a total of 96 trials, 64 in which the experimenter asked to give a shock and 32 in which the experimenter asked not to give a shock, as in^38^.

To be able to measure the neural response to the pain of others, volunteers were told to always look at the hand of the victim during the experiment and to move as little as possible. To ensure that participants were actually looking at the real-time video showing the victim’s hand, on some trials, they were asked to rate how painful the shock delivered to the victim was. Another analogue scale then appeared on the screen, ranging from ‘*not painful at all*’ (0) to ‘*very painful*’ (1000), again with a cursor starting at a random position on that scale.

For both groups the experimenter was a Caucasian-type, female experimenter. A former study^64^ indicated that it is important that participants recruited in Rwanda do not think that the experimenter is affiliated with the Rwandan government. For this reason, we did not use a Rwandan experimenter to give orders to the participants tested in Rwanda.

If some volunteers would have decided to act in a fully prosocial fashion by systematically refusing to administer shocks to the victim, this would have led to no EEG data associated with the visualization of the electric shock sent to the victim’s hand. Therefore, the session ended with an additional observation block, during which volunteers visualized 14 trials with a shock sent to the victim’s hand and 14 trials with no shock. A tone was again systematically played. Volunteers were told to always look at the hand of the victim during that block and that the computer will send or not send the shock. To obtain a similar predictability of outcomes between the observation block and the main experimental block, a square labelled either SHOCK or NO SHOCK was displayed on the screen at the beginning of the trial. Volunteers were told that they would still have to complete the pain scale, similarly to the main experimental block.

At the end of the experimental session, participants were asked to fill in six questionnaires presented in both English and Kinyarwanda for the group tested in Rwanda and in French for the group tested in Belgium. For the translation in Kinyarwanda, a back translation procedure was used. A bilingual research assistant first translated each item in Kinyarwanda. Then, a professor (second author of this paper) translated back to English each item. They then discussed the potential discrepancies and ensured that they agreed on the translation of specific words that can be culturally different in the two languages. Those questionnaires included (1) the Money Attitude Scale^52^, (2) the Moral Foundation Questionnaire^51^, (3) the Aggression-Submission-Conventionalism scale^49^, (4) a debriefing assessing what they felt during the experiment and the reasons for choosing to obey or disobey the orders of the experimenter (see Supplementary Material S2 for the questionnaire in English and Kinyarwanda), (5) a questionnaire assessing the family trauma during the genocide and the participation to reconciliation programs (only for the group tested in Rwanda) (see Supplementary Material S3 for the questionnaire in English and Kinyarwanda) and (6) a questionnaire on social identification to the experimenter^12^, see Supplementary Material S4 for the questionnaire in English and Kinyarwanda). We could not ask participants in Rwanda for their ethnic group and to what extent they considered their co-participant as part of their own group or not because it is illegal to mention ethnicity or differences between ‘groups’ of individuals in Rwanda.

### EEG recordings

Brain activity was recorded using a 32-channel electrode cap with the ActiveTwo system (BioSemi) and data were analyzed using Fieldtrip software^56^. The activities from left and right mastoids and from horizontal and vertical eye movements were also recorded. Amplified voltages were sampled at 2048 Hz. Data were referenced to the average signal of the mastoids and filtered (low-pass at 40 Hz and high-pass at 0.01 Hz). To clean the data from the line noise we then used a spectral interpolation around 50 Hz and its harmonics. Artefacts due to eye movements were removed with an Independent Component Analysis (ICA). A final cleaning based on visual inspection was then performed. 3.9% of the trials were removed. Because of the EEG recordings, participants were further instructed to wait a minimum of 1 s in a relaxed position before pressing a key, so as to obtain a consistent and noise-free baseline taken − 500 to − 300 ms before the occurrence of the tone. Participants were additionally instructed not to move for up to 2 s after the tone and asked to avoid blinking when they pressed a key. To ensure that participants respected the 2 s without moving and blinking after the tone, we told them to wait for the time estimation scale to appear on the screen as a cue.

All event-related potentials in the time-domain were analyzed across Fz, Cz and Pz, similar to past studies^57,58^. The time windows of the ERP components were chosen according to visual inspection of the grand-averaged data similar to past studies^57,59,60^ as well as prior knowledge. The N1 and the N2 were measured as the most negative peaks within the 30–130 ms time window and the 240–340 ms time window, respectively, after the tone. The P2 and the P3 were measured as the most positive peak within the 130–230 ms time window and the 340–440 ms time window, respectively, after the tone. The early LPP and the late LPP were measured as the mean amplitude between the 440–650 ms time window and the 650–900 ms time window, respectively, after the tone.

Based on previous studies, we measured the frontal midline theta frequency (FMθ) over the Fz, Cz, FC1 and FC2 electrodes. We extracted the time–frequency power of each trial after calculating a Fast Fourrier transform (FFT) on our data and an FFT of a complex Morlet wave with the following parameters: frequency from 2 to 30 Hz with 80 bins spaced logarithmically and 4–14 cycles logarithmically spaced. Then, we used the Inversed Fast Fourrier Transform (IFFT) method on the multiplication of the computed FFT on our data and the FFT of the complex Morlet Wave. Epoching was performed by taking − 3 to 1s around the keypress (response-locked time window) and − 1 to 3 s around the presentation of the auditory instruction (stimulus-locked time window). All power values in the time–frequency representation were normalized to the average pre-stimulus baseline power at each frequency band. We used a decibel (dB) transform for normalization [dB power = 10 × log_10_(power/baseline)]. The baseline power was computed as the average power across all experimental conditions, from 300 to 500 ms after the keypress when TFR were response-locked and from − 200 ms to 0 ms when TFR were stimulus-locked.

## Results

Results were analyzed with both frequentist and Bayesian statistics in order to provide complementary evidence in favor of H_1_ or H_0_^61^. Supplementary Material S5 provides more information on Bayesian statistics and how they were computed in the present study. Data deviating from more than 2 standard deviations were excluded.

### Prosocial disobedience

**Figure 2:**
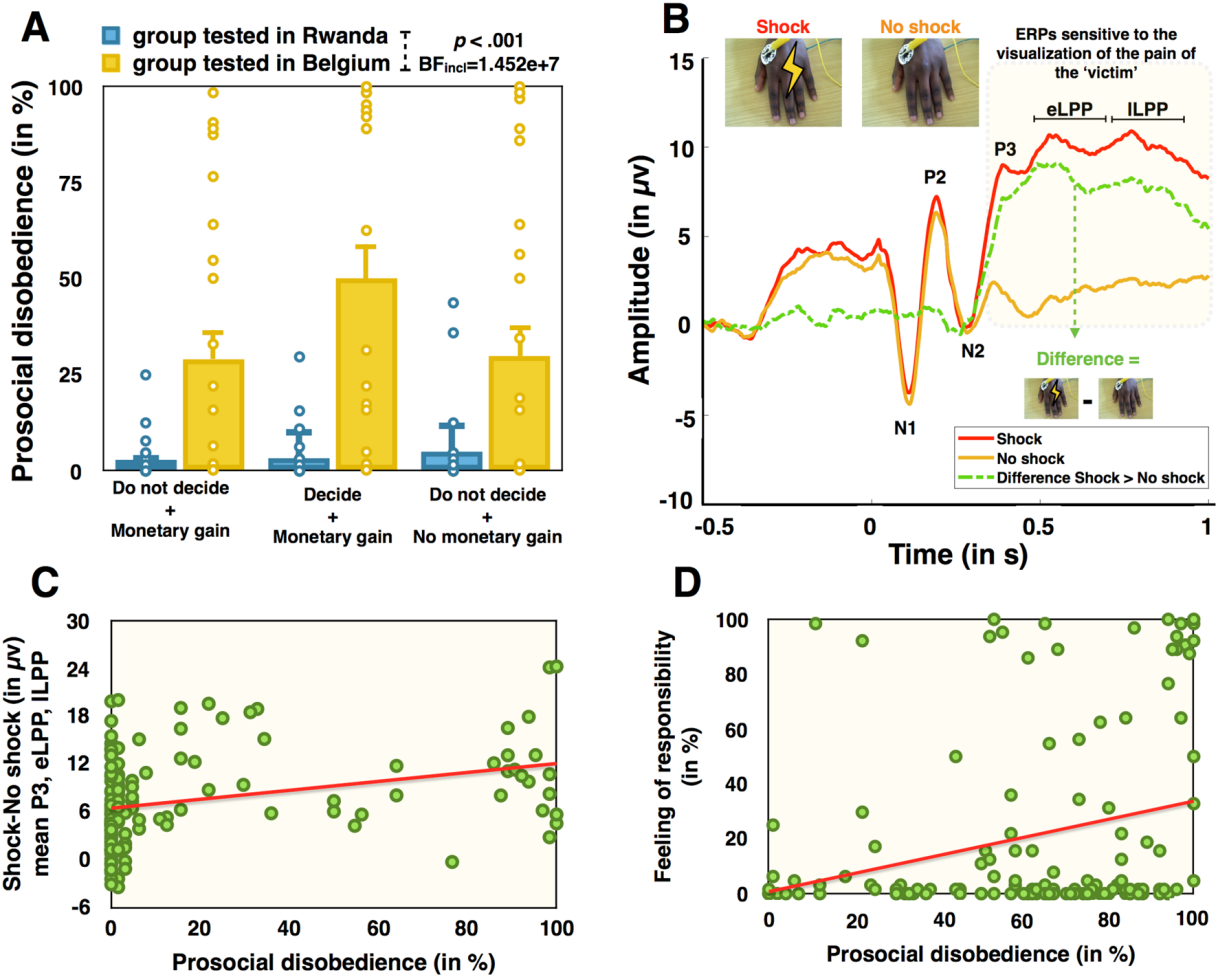
(**A**) Prosocial disobedience (in %) in the group of Rwandese tested in Rwanda (in blue) and in the group of Rwandese tested in Belgium (in yellow) across the three variants of the task. Colored dots represent individual data. Prosocial disobedience was higher for the group tested in Belgium than for the group tested in Rwanda in the three variants. Error bars represent standard errors. All tests were two-tailed. (**B**) Grand averaged waves from all participants. The red line represents the waves when participants heard the tone and visualize a shock on the hand of the victim. The orange line represents the same waves when participants heard the tone but did not visualize a shock on the victim’s hand. The dotted green line represents the difference between the two waves. (**C**) Graphical representation of the correlation between the neural processing of pain in the observation block and prosocial disobedience. (**D**) Graphical representation of the correlation between the feeling of responsibility and prosocial disobedience.

We calculated the percentage of prosocial disobedience for each participant, corresponding to the number of trials participants chose to disobey (i.e., sending no shocks while ordered by the experimenter to do so) by the total number of trials corresponding to the order to send a shock, multiplied by 100. We compared the prosocial disobedience rate across groups and variants of the task. We conducted a univariate ANOVA with prosocial disobedience as the dependent variable and Group (Rwandese tested in Rwanda, Rwandese tested in Belgium) and Variant of the Study (Do not decide + Monetary gain; Decide + Monetary gain; Do not decide + No monetary gain) as fixed factors. Both frequentist and Bayesian approaches indicated a main effect of Group (F_(1,138)_ = 45.963, *p* < .001, η^2^_*partial*_ = .250, BF_incl_ = 1.452e +7), with higher prosocial disobedience rates in the group tested in Belgium (36.26%, CI_95_ = 29.5–43) compared to the group tested in Rwanda (3.66%, CI_95_ =  − 3.05 to 10.39), see Fig. 2A. The main effect of Variant and the interaction were slightly in favor of H_0_ (all *ps* > .1 and all BFs_incl_ < .625). We replicated the same univariate ANOVA with Gender as an additional fixed factor. The main effect of Gender was in favor of H_0_ (*p* > .4, BF_incl_ = .269) and the pattern of results remained unchanged. We also conducted additional control analysis that showed that order of the role (victim first, agent first) did not influence the results (*p* > .2, BF_incl_ = .331). Of note, 9/72 participants in the group tested in Rwanda and 4/72 participants in the group tested in Belgium disobeyed in an antisocial way, that is, they administered a shock to the ‘victim’ even if the experimenter asked not to do so.

### Neural processing of the victim’s pain

We compared the neural processing of pain when participants witnessed a shock being delivered on the hand of the ‘victim’. Based on both previous literature and the visual inspection of the grand averaged waves, we extracted the amplitude of several event-related potentials (ERPs) associated with either auditory outcome processing (N1, P2) or with pain outcome processing (N2, P3, eLPP, lLPP^57^), see Fig. 2B (see Materials and Methods—EEG recordings). We first compared the amplitude of those ERPs when participants witnessed a shock on the victim’s hand *vs* when they did not witness a shock, in order to identify ERPs sensitive to the visualization of pain. Results indicated a higher amplitude for Shock trials *vs* No shock trials for the P3, the eLPP and the lLPP. No evidence was found for this difference on the N1, P2 and N2 (see Supplementary Material S6 for full results).

We then subtracted the amplitude of those potentials during No shock trials to Shock trials (i.e., Shock-No shock) and performed statistical comparison between the two groups on this new variable. We did not observe conclusive evidence for a difference in the neural processing of pain between the group tested in Rwanda and the group tested in Belgium (all *p*s ≥ .018, all BFs_10_ ≥ .186 and ≤ 2.426). As a reminder, behavioral results indicated that the group tested in Belgium refused to administer a shock to the ‘victim’ more frequently compared to the group tested in Rwanda. Consequently, it could have led to a reduced amplitude of pain-related potentials due to a higher repetition suppression (RS). We thus performed similar analyses on the observation block, i.e., an additional block that was run at the end of the experiment for each participant (see *Supplementary Material S7* for Shock-No Shock comparisons). Since the frequency of shock and no shock trials was controlled in the observation block, RS was controlled. Results in the observation block were similar to the main experimental block with overall no conclusive difference between the two groups (all *p*s ≥ .017, all BFs_10_ ≥ .331 & ≤ 2.601). Only the amplitude of the P3 was higher in the sample tested in Belgium than in the group tested in Rwanda (t_(135)_ =  − 2.657, *p* = .009, Cohen’s d =  − .454, BF_10_ = 4.371). Additional control analysis showed that order of the role (victim first, agent first) did not influence the results for all ERPs (all *ps* > .1).

Since overall results were similar between the main experimental block and the observation block but only the observation block allowed to include participants who disobeyed the most, subsequent analyses were conducted on ERPs in the observation block. We computed a general pain response by averaging the amplitude of the P3, eLPP and lLPP and used that pain response as the predictor variable into the model, together with the N1, P2 and N2. Results indicated that only the neural processing of pain was a reliable predictor of prosocial disobedience (t_(136)_ = 4.072, *p* < .001, Beta = .446, BF_incl_ = 109.749), with a higher neural response to the pain of the ‘victim’ being associated with a higher percentage of prosocial disobedience, see Fig. 2C. Interestingly, that pain response positively correlated with the subjective pain ratings (r = .275, *p* = .001, BF_10_ = 20.267), indicating that those with a higher neural response to the pain of the victim also estimated the pain of the victim as higher. We also ensured that the neural processing of pain was not stimulus-dependent, i.e., relative to individual differences between victims (see *Supplementary Material S8*). Other ERPs (i.e., N1, P2 and N2) were inconclusive (all *ps* < .1, all BFs_incl_ ≥ .405 and ≤ .904). All VIFs were below 2.5.

### Sense of agency and feeling of responsibility

As a reminder, the sense of agency was measured through a proxy based on interval estimates. Because interval estimates were planned to be correlated with prosocial disobedience, we first transformed the raw interval estimates in z-score data. It is indeed known that participants may differ in the way they use the ms-scale to provide an answer, some preferring smaller numbers and others preferring larger numbers^28,62^. Z-scores reduce irrelevant inter-subject variability by subtracting from each interval estimate, the mean estimate for that participant across all trials and by dividing the resulting differences by the standard deviation of all estimates for that participant. The z-scored interval estimates are interpreted as the raw interval estimates are, with lower z-scored interval estimates being interpreted as a higher sense of agency (SoA). Independent sample t-tests conducted on z-scored interval estimates when participants obeyed to send a shock to the ‘victim’ did not differ between groups (*p* > .8, BF_10_ = .263). A similar comparison on trials where participants obeyed not to send a shock to the ‘victim’ was inconclusive (*p* > .07, BF_10_ = 1.031). Comparisons in z-scored interval estimates between obedience and disobedience trials were unreliable, since they included only N = 9/72 in the group tested in Rwanda and N = 23/72 in the group tested in Belgium. This small sample was the results of the low rate of disobedience in the group tested in Rwanda combined with the failure to realize the task of interval estimates correctly. We further observed that the two groups did not differ in how responsible they felt for the outcomes of their actions during the experiment (*p* > .5, BF_10_ = .207).

We then conducted a multiple linear regression with the feeling of responsibility, z-scored interval estimates for shock trials when participants obeyed, and z-scored interval estimates for no shock trials when participants obeyed as the independent variables and prosocial disobedience as the dependent variable. Results indicated that how responsible participants felt during the task was the best predictor of prosocial disobedience (t_(63)_ = 3.397, *p* = .001, Beta = .395, BF_incl_ = 35.511). We observed that a higher reported feeling of responsibility during the task was associated with a higher prosocial disobedience, see Fig. 2D. Z-scored interval estimates for shock trials or no shock trials when participants obeyed were in favor of H_0_ (*p* > .1, BF_incl_ = .323 and *p* > .8, BF_incl_ = .909, respectively). All VIFs were below 2. Of note, a similar result was obtained for how responsible participants felt during the task when the whole sample was considered – and not only those who successfully performed the interval estimates task (t_(143)_ = 3.384, *p* < .001, Beta = .273, BF_incl_ = 29.84).

### FMθ after the auditory instructions

**Figure 3:**
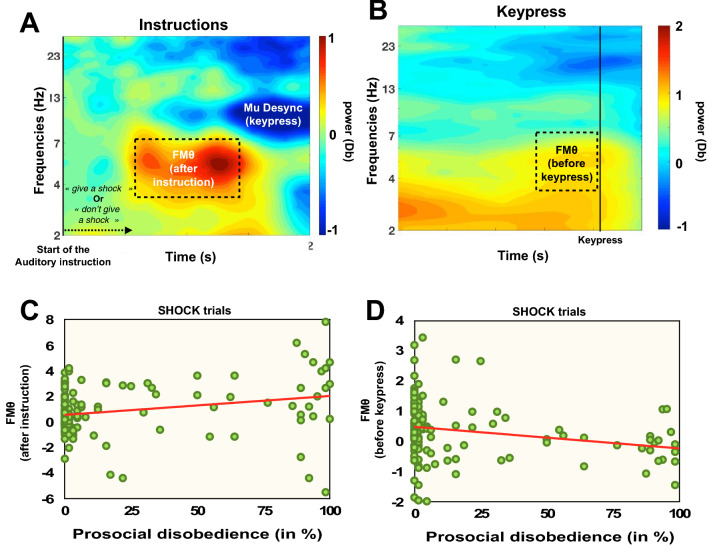
Time frequency power plots for the 4 electrodes (Fz, Cz, FC1, FC2) after hearing the instructions (**A**) and before pressing the key to obey the orders of the experimenter (**B**). Mu D represents the desynchronization in the Mu band (8-12 Hz), which is typical of movement execution (i.e., keypress). (**C**) Graphical correlation of the positive correlation between FMθ after the instruction to send a shock to the victim and prosocial disobedience. (**D**) Graphical correlation of the negative correlation between FMθ before the keypress associated with sending a shock to the victim and prosocial disobedience. All tests were two-tailed.

Based on visual inspection of the grand average data (see Fig. 3A), FMθ was extracted from a 460–1.580 ms time window after the auditory instructions in the 3.4–7.2 Hz frequency bands. We systematically extracted the mean value in this time/frequency window for each participant. We conducted a repeated-measures ANOVA with Instruction (send a shock; do not send a shock) as within-subject factors and Group (Rwandese tested in Rwanda, Rwandese tested in Belgium) and Variant of the Study (Do not decide + Monetary gain; Decide + Monetary gain; Do not decide + No monetary gain) as between subject factors on the FMθ after hearing the auditory instructions. We observed a strong evidence in favor of H_1_ for a main effect of Group (F_(1,124)_ = 29.249, *p* < .001, η^2^_*partial*_ = .191, BF_incl_ = 16308.42). Results indicated that FMθ was higher for the group of Rwandese tested in Rwanda when they received instructions (.87; CI_95_ = .69–1.06) than for the group of Rwandese tested in Belgium (.11; CI_95_ =  − .09 to 0.32). We also observed a main effect of Instruction with the frequentist approach (F_(1,124)_ = 6.441, *p* = .012, η^2^_*partial*_ = .049), with more FMθ after hearing the instruction not to send a shock than after hearing the instruction to send a shock. However, the same result was inconclusive with the Bayesian approach (BF_incl_ = 1.271). Other interactions were inconclusive or in favor of H_0_ (all *ps* < .3, all BFs_incl_ ≥ .015 and ≤ .425). Additional control analysis showed that order of the role (victim first, agent first) did not influence the results (*p* > .7, BF_incl_ = .196). Pearson correlations indicated that the lower the FMθ after hearing the order to send a shock to the victim was, the higher the prosocial disobedience rate was (r =  − .258, *p* = .002, BF_10_ = 10.282), see Fig. 3C.

As we hypothesized that distancing oneself from the authority would involve a lower attention to the orders given by that authority, we conducted exploratory analyses to correlate the FMθ and the different self-reported questionnaires evaluating one’s own relationship to authority (e.g., the submission subscale of the ASC questionnaire; the authority subscale of the ASC questionnaire; the 3 subscales of the questionnaire regarding the identification with the experimenter). Results indicated that a higher FMθ when hearing the order to send a shock was associated with higher scores to the submission subscale (r = .251, *p*_FDR_ = .006; BF_10_ = 6.566), a higher score to the authority subscale (r = .282, *p*_FDR_ = .005; BF_10_ = 20.478) and a higher score to bonding with the experimenter (r = .262, *p*_FDR_ = .006; BF_10_ = 9.772). None of the correlations were significant with the instructions not to send a shock (all *p*s > .1, all BFs_10_ < .794).

### FMθ before the keypress

Based on visual inspection of the grand average data (see Fig. 3B), FMθ was extracted from a (−).540 to (−).080 ms time window before the keypress in the 4–6.4 Hz frequency bands. We systematically extracted the mean value in this time/frequency window for each participant. We conducted a repeated-measures ANOVA with Shock (Shock sent; No shock sent) as a within-subject factor and Group (Rwandese tested in Rwanda, Rwandese tested in Belgium) and Variant of the Study (Do not decide + Monetary gain; Decide + Monetary gain; Do not decide + No monetary gain) as between-subject factors on the FMθ before obeying to orders. Of note, we did not integrate the condition when participants disobeyed as there were no enough participants who disobeyed in the group tested in Rwanda in each variant (N = 4 in variant1; N = 7 in variant 2; N = 7 in variant 3). We observed a main effect of Group (F_(1,126)_ = 11.260, *p* = .001, η^2^_*partial*_ = .084, BF_incl_ = 10.732), with more FMθ in the group tested in Belgium (1.43, CI_95_ = 1–1.8) compared to the group tested in Rwanda (0.45, CI_95_ = .07–0.83). Additional control analysis showed that order of the role (victim first, agent first) did not influence the results (*p* > .4, BF_incl_ = .195). Pearson correlations indicated that the higher the FMθ was before obeying the order to send a shock to the victim, the higher the prosocial disobedience rate was (r = .301, *p* = .001, BF_10_ = 42.879), see Fig. 3D.

As each shock in Variants 1 and 2 was associated with a monetary reward, we conducted exploratory analysis between the FMθ before sending or not a shock to the ‘victim’ and the different subscales of the MAS questionnaire measuring one’s attitude to money. Results indicated that a higher score on the retention subscale of the MAS was associated with a lower FMθ before sending a shock to the victim (r =  − .273, *p*_FDR_ = .012; BF_10_ = 3.119). Other correlations with FMθ before not sending a shock were not statistically associated with the different subscales (all *p*s > .083, all BFs_10_ < .591).

### Self-reported personality traits and identification to the experimenter

We also conducted exploratory analyses on the cultural, social and psychological dimensions that could differ between the two groups and that could be associated with prosocial disobedience. Supplementary Material S9 and Table S1 display the statistical comparisons between the group tested in Rwanda and the group tested in Belgium for the different social and individual dimensions (i.e., relationship to authority, moral foundations, money attitude, identification with the experimenter). For each of those dimensions, we conducted separate multiple linear regressions in order to identify the subscales that were associated the most with prosocial disobedience. We performed the analyses on the subscales instead of the overall scores to each questionnaire as each subscale reflect a different theoretical construct. Overall, results showed that prosocial disobedience was reliably negatively associated with the submission subscale (ASC questionnaire) and reliably positively associated with the harm subscale (Moral Foundation Questionnaire).

### Family trauma and participation in reconciliation activities

Full results are displayed in Supplementary Material S10. Overall, results indicated that participants who disobeyed the most frequently were also those who indicated a higher family suffering during the genocide. How frequently participants talked about those events with their family, how much participants think that their family history during the genocide influence their behaviors and how frequently they attended reconciliation activities organized on a yearly basis did not reliably influence prosocial disobedience.

### Multiple linear regression

In exploratory analyses, we run a multiple linear regression including all main factors influencing prosocial disobedience observed in the above-mentioned analyses in order to evaluate the respective weight of each of those variables on prosocial disobedience. Those variables included the following: the ERPs associated with the processing of pain, how responsible participants felt during the task, FMθ after hearing the instructions to send a shock to the victim, FMθ before obeying the orders to send a shock to the victim, the reported family suffering during the 1994 genocide, the scores on the submission subscale and the scores on the harm subscale. Predictor variables for questionnaires were centered before building the model. To mitigate the risk of false discovery with the frequentist approach, we applied a False Discovery Rate (FDR) approach with the Benjamini and Hochberg method to each p-value. As only 10/72 participants disobeyed in a prosocial way in the group tested in Rwanda, we did not have sufficient power to perform such analyses on each group separately. The results were thus performed on the two groups of participants together. Table 1 displays the full results, from the most reliable predictor on the top to the less reliable predictor on the bottom based on the Beta values. All VIFs were below 2, suggesting an absence of collinearity.

**Table 1:** Predictors of prosocial disobedience.

	t	df	Beta	*p*	BF_incl_
Feeling of responsibility	4.107	121	.320	< .001	764.22
FMθ before sending a shock	4.058	121	.312	< .001	1253.42
Submission subscale	− 2.775	121	− .221	.006	34.73
Neural response to the victim’s pain	2.081	121	.164	.04	7.03
Estimated family suffering	1.551	121	.122	> .1	3.38
Harm subscale	1.020	121	.080	> .3	1.74
FMθ after instruction to send a shock	− .912	121	− .072	> .3	1.58

### Reasons for disobeying

Additional analyses were conducted on the reasons that participants gave to explain why they disobeyed or obeyed the orders of the experimenter and how it related to prosocial disobedience. The full results and individual statements are presented in Supplementary Material S11. Overall, results indicated that the percentage of prosocial disobedience positively correlated with moral reasons. Results also indicated that the more agents felt bad for the ‘victim’, the higher the prosocial disobedience was. We further observed that the less participants were interested in making money, the more they refused immoral orders. Exploratory analyses on antisocial disobedience are reported in Supplementary Material S12.

## Discussion

In the region of the Great Lakes in Africa, collective victimhood and a desire for revenge have been considered as key elements explaining the ongoing conflicts^63^. The Genocide Against Tutsis represents the culmination point of such collective victimhood in Rwanda^47^. A key societal question is to understand what influences the decision of the new generations in war-torn societies to persevere in destructive behaviours. In the present study, we examined the specific situation of obedience to orders to inflict harm to another individual.

We targeted several neuro-cognitive processes in order to investigate the temporal dynamics of disobedience to immoral orders. Results indicated that the more participants processed the auditory instruction to send a shock to the ‘victim’, as indexed by a higher FMθ, the lower prosocial disobedience was. Previous studies suggested that a high FMθ during the processing of auditory stimuli is associated with a greater allocation of attentional resources^10,11^. This could indicate that disobedience is facilitated when individuals disengage their attention from the orders received by the authority. This disengagement could be facilitated by a low relationship to authority, as we observed that people scoring low to the different questionnaires assessing their relationship to authority also had a lower FMθ. As individuals in the group tested in Rwanda scored systematically higher regarding their relationship to authority compared to the group tested in Belgium, this may explain why FMθ was higher in the group tested in Rwanda, as well as obedience. This result also highlights that culture has an important weight when explaining people’s compliant behaviors. Interestingly, we observed a higher FMθ after hearing the auditory instruction in the group tested in Rwanda compared to the group tested in Belgium. As the group tested in Rwanda also had a lower prosocial disobedience rate compared to the group tested in Belgium, this would further support that disengaging from the orders of the authority facilitates disobedience.

We observed a higher FMθ before obeying to send a shock to the ‘victim’ in the group tested in Belgium compared to the group tested in Rwanda. According to past literature, which showed that a stronger conflict elicits a higher activity in FMθ compared to low conflicts^17–19^, it may thus suggest that obeying the orders to send a shock to the victim was perceived as an action inducing a stronger conflict in the group tested in Belgium compared to the group tested in Rwanda. Critically, this result could also explain why prosocial disobedience was higher in the group tested in Belgium compared to the group tested in Rwanda. We indeed also observed that a higher FMθ before obeying to send a shock to the ‘victim’ was associated with a higher prosocial disobedience rate. Interestingly, exploratory analyses showed that scoring high to the retention subscale of the money attitude scale was associated with a lower FMθ before sending a shock. This result may indicate the aversion to hurt others may be attenuated with the obtention of a financial reward for the pain inflicted, especially if the importance given to money is high.

We observed that a higher feeling of responsibility was associated with a higher prosocial disobedience rate. This result supports previous studies showing that feeling responsible has a positive influence on prosocial behaviours^31,64,65^. The lack of data regarding interval estimates, used as a proxy for the sense of agency, precluded from studying with a sufficient statistical power the relationship between prosocial disobedience and the sense of agency. Critically, we indeed had to exclude a larger number of participants in the interval estimate task due to a failure to discriminate between millisecond intervals compared to previous studies^66^. This unexpected difficulty is unlikely to be due to comprehension since all participants were explained the task in their native language. It is also unlikely that participants were not motivated to perform the task correctly since they largely reported a strong willingness to help researchers to obtain accurate data. One explanation could be related to different processing strategies in different cultures, since former samples were composed in majority of western individuals^67^. However, this possibility is only an assumption as it has never been investigated in the scientific literature.

We also observed that a higher neural response to the pain of others was associated with a greater prosocial disobedience. Many studies have shown that feeling empathy for the pain of others has a positive influence on prosocial behaviors^31,34,35^, as the sharing of vicarious pain prevents aggression and promotes help^68,69^. The present study indicates that empathy for pain could also support the possibility to resist orders to hurt another person.

We further observed that people scoring high to the harm subscale of the moral foundation questionnaire had a higher prosocial disobedience rate. The harm subscale is theoretically defined as being related to our evolution, which involved an ability to feel and dislike the pain of others^51^. It thus suggests that the more people feel aversion for the pain of others, the more likely they will refuse orders to inflict pain to another person.

Finally, we observed that the more their family suffered during the 1994 genocide, the more this first generation would resist immoral orders. A possible interpretation to this effect is that individuals coming from families that were strongly impacted by the genocide would be less likely to perpetrate acts of obedience, which are described as a critical factor explaining the genocide^40,41^. We nonetheless observed that a higher reported family suffering was also associated with a higher neural response to the pain of others. Other interpretations are thus possible. On the one hand, it may be the case that a high family suffering involved a higher sensibilization to the suffering of others^70,71^, thus leading to more prosocial behaviors. One the other hand, people having a higher neural response to the pain of others could also estimate the suffering of their own family as higher. Future research is thus necessary to better understand the link between family suffering during a genocide and obedience to immoral orders.

**Figure 4:**
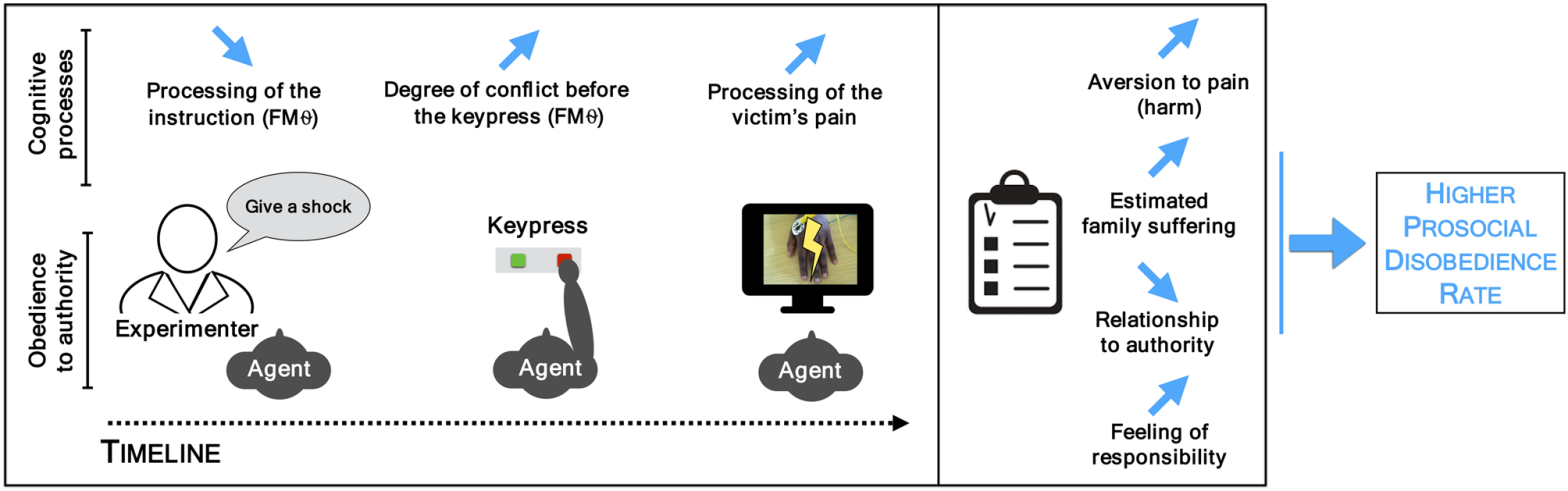
Schematic representation of the different factors associated with a higher prosocial disobedience rate. The blue line indicates the directionality of the association.

Altogether, those results suggest that several neuro-cognitive processes occurring at different moments during an act of obedience to immoral orders can influence prosocial disobedience. As a resume, we observed that a low processing of the instructions of the experimenter, a high degree of conflict before obeying the orders to send a shock to the victim, a higher neural response to the pain of others and a high feeling of responsibility were associated with a higher prosocial disobedience rate (see Fig. 4). Caution should nonetheless be taken regarding the feeling of responsibility in our study as it relied on a single estimation at the end of the block. It may also be the case that individuals sending more shocks would tend the under-report their perceived responsibility afterwards in order to maintain a positive self-image^72^. Importantly, those effects could also be influenced by the cultural relationship to authority and the reported family suffering during the genocide. Our study thus revealed critical cognitive factors involved in prosocial disobedience. However, due to the lack of past literature, the precise influence of each of those factors on prosocial disobedience and to what extent those factors are mutually exclusive or inclusive should still be determined.

We did not observe a significant difference in prosocial disobedience between the different variants of the same task. The presence or the absence of the monetary gain did not influence the results. This result could be explained by the amount of money per se (i.e., + €0.05/ + RWF50); a higher monetary reward would have maybe led to a greater difference in obedience. However, in a former study with a similar paradigm, offering a monetary reward or not did influence prosocial disobedience^38^. One possibility is that the populations between this previous study and the present study were highly different. While the population tested in Caspar^38^ was composed of western individuals, the population tested in the present study was composed of individuals with a family and national history strongly influenced by the genocide. It may thus be the case that social and individual dimensions supporting disobedience differ between these two populations. This also underlies the importance for social neuroscience research to avoid studying only WEIRD (western, educated, industrialized, rich and democratic) populations in their sample in order to broaden its conclusions to more diverse human beings. We also observed that explicitly offering participants the possibility to disobey did not influence the results in variant 2. This may reflect a general tendency of following authorities as a personal choice.

A previous study conducted in Rwanda indicated that listening to peace building radio programs did not necessarily involve a cultural change in deference to authority, but induced changes in behaviors, with listeners being more likely to defer to local officials for the resolution of problems^73^. However, that study only relied on fictitious scenarios presented through questionnaires, thus preluding the possibility to investigate behavioral changes. In our study, the frequency to which individuals participate in reconciliation activities did not influence prosocial disobedience. A critical difference is that none of our participants were reminded about such peace messages prior to their participation in the study.

In the present study, we did not directly assess whether or not our participants knew about the experiments of Stanley Milgram. One could expect that knowing Milgram would prevent people to obey and that it could account for differences between groups. In the present study, we asked each of our volunteers if they disobeyed because they thought it was the main of the study, thus indirectly targeting Milgram’s studies on disobedience. Results indicated that the two groups did not differ in their ratings on this question. Further, ratings on this question were statistically not associated with prosocial disobedience. Those results confirmed a previous study^38^ and suggest that thinking that disobedience is the main aim of the study is not statistically associated with prosocial disobedience.

We acknowledge that our study lacks a critical factor that would control for in-group or intergroup attitudes within our pairs of Rwandese participants. For legal and ethical reasons in Rwanda, variables related to ethnicity cannot be measured^74^. Also, the political system strongly differs between Belgium and Rwanda, an aspect that could influence or reflect a general compliance with the hierarchy.

In the present study, we also had a confound between site of the experiment (i.e., Rwanda or Belgium) and Gender, as we failed to recruit an equal number of female and male participants in Rwanda. However, results indicated that prosocial disobedience was not influenced by gender. These results are consistent with Milgram’s studies showing no difference between men and women^4^ and with a previous study showing that the effect of Gender on prosocial disobedience was inconclusive^38^.

As our design implied a role reversal procedure, one could expect that the role of the role has an effect on the results. However, statistical analyses showed that order of the role never conclusively influenced the results, which is consistent with past studies using a relatively similar procedure^27,29,38,75^.

Future studies could also refine the questions asked regarding the family trauma. For instance, regarding the questions related to the number of family members who died and suffered during the genocide, we realized that those were very difficult questions to answer for the majority of volunteers, and for several reasons. First, it is common in Rwanda for individuals not to know precisely what happened to their relatives during the genocide. Second, several volunteers reported that they only very rarely talked with their relatives about what happened in 1994. The topic remains sensitive for the majority of families, which avoid speaking about the genocide.

The present study is one of the first that investigated the neuro-cognitive processes and the temporal dynamics at the neural level, together with cultural, social and psychological factors, that are associated with prosocial disobedience. The lack of previous literature on such question is probably due to the fact that the scientific community largely avoided to conduct research on disobedience after the ethical concerns associated with Milgram’s studies^37^. Subsequent variants were adapted regarding ethical standards^5,6^, but were not adapted to neuro-cognitive measurements. There is thus still a long path to understanding the mechanisms supporting prosocial disobedience. As we see in the present study, several factors can influence individual decisions to disobey orders to harm another person, which show the complexity for understanding better human behaviors.

In conclusion, it is important to remember that there is a significant gap between obeying immoral orders in an experimental set-up and participating in a massacre. The significant prevalence of obedience behaviors in a sample tested in Rwanda does not necessarily imply causality with the genocide. A range of determining contextual factors also have to be considered, such as economic conditions^76^ or dehumanization^77^. For instance, as the pain induced in our experimental set-up did not involve any long-term effects, we advise against conducting inferences about the extent to which the results observed predict or reflect similar behavioral conduct in real life. Moreover, participants in this study are not likely to represent the population that committed the genocide. The aim of the present study was to identify factors that influence prosocial disobedience, by suggesting that perhaps those factors also contribute to prosocial disobedience in real life.

## Supplementary Information

Supplementary Information.

## Data Availability

Data and codes are available on Open Science Framework (https://osf.io/hmzy8/).
