# Supplementary material for: On the cognitive mechanisms supporting prosocial disobedience in a post-genocidal context
Source: Sci Rep. 2022 Dec 19;12:21875. doi: 10.1038/s41598-022-26460-z (PMC9763397; doi:10.1038/s41598-022-26460-z)
Supplement: Supplementary file 1 — Supplementary Information. [file 41598_2022_26460_MOESM1_ESM.docx]

Supplementary Materials for

**On the cognitive mechanisms supporting prosocial disobedience in a post-genocidal context**

***Supplementary Material S1***

***Variants “Do not decide + (no) monetary reward”***

*In this experiment, you will get paid for your participation RWF20,000, no matter your decisions during the task.*

*I, ………………… understand that I will be paid RWF20,000 for my participation in this experiment, no matter my decisions during the task.*

*I declare that I have understood the above-mentioned instructions.*

*Date: ……………*

*Signature: ……………*

***Variant “Decide + monetary reward”***

*In this experiment, you will get paid for your participation RWF20,000, no matter your decisions during the task.*

*I, ………………… understand that I will be paid RWF20,000 for my participation in this experiment, no matter my decisions during the task.*

*I also understand that I am free to make my own decisions, therefore to decide whether to inflict or not a shock to the victim in exchange for RWF50.*

*I declare that I have understood the above-mentioned instructions.*

*Date: ……………*

*Signature: ……………*

***Supplementary Material S2***

**Please put a trait on the lines to describe your experience. It refers to the situation when you were AGENT ONLY. *Translation:* *Shyira akamenyetso ku gipimo cy’umurongo wahawe werekane ibyakubayeho igihe wari mu bushakashatsi mu gihe wasabwaga kohereza amashanyarazi muri mugenzi wawe ukorerwaho ubushakashatsi***

1. How responsible did you feel when you were the agent? *Translation: Ni ku ruhe rugero wumvise bibaye inshingano zawe igihe wasabwaga kohereza imbaraga z’amashanyarazi muri mugenzi wawe?*

Not at all Extremely

Nta na rimwe Birenze/mu buryo bukabije

2. How bad did you feel when you delivered a shock in exchange of money? *Translation:Ni ku kihe kigero wumvise ubabaye igihe woherezaga imbaraga z’amashanyarazi mu wundi muntu*

Not at all Extremely

Nta na rimwe Birenze/mu buryo bukabije

3. How sorry did you feel when you delivered shocks to the ‘victim’? *Translation:Ni ku kihe kigero wababaye/wicujije igihe woherezaga imbaraga z’amashanyarazi mu wundi muntu*

Not at all Extremely

Nta na rimwe Birenze/mu buryo bukabije

4. Please rate how painful do you think the shocks were for the victim? *Translation:* *Gerageza ushyire ku gipimo ububabare watekerezaga ko uri guha mugenzi wawe igihe wamwoherezagamo imbaraga z’amashanyarazi?*

Not at all Extremely

Nta na rimwe Birenze/mu buryo bukabije

5. Did you intentionally disobey? *Translation:* *Ese wigeze wanga gukora ibyo usabwa ku bushake?*

**YES NO**

***YEGO OYA***

- If you indicated **NO** in the previous question, can you explain your reasons for following the orders throughout the experiment? *Translation:* *Niba ari oya ni dusobanurire impamvu yatumaga ukurikiza amategeko wahabwaga mu mwanya wamaze mu bushakashatsi?*

__________________________________________________________________________________________________________________________________________________________________________________________________________________________________________________________________________________________________________________________________________________________________________________________________________________________________________________________________

- If you indicated **YES** in the previous question, please answer the following questions about the reasons for disobeying. *Translation: Niba ari* ***YEGO*** *subiza ibi bibazo bikurikira*

A. I felt bad for the victim. *Translation:* *Numvaga bimbabaje kohereza imbaraga z’amashanyarazi muri mugenzi wanjye*

Not at all Extremely

Nta na rimwe Birenze/mu buryo bukabije

B. There were too many shocks. *Translation:* *Nasabwaga kubikora inshuro nyinshi*

Not at all Extremely

Nta na rimwe Birenze/mu buryo bukabije

C. I don’t like being told what to do. *Translation:* *Ntago nkunda abambwiriza ibyo gukora*

Not at all Extremely

Nta na rimwe Birenze/mu buryo bukabije

D. I wanted to make more money. *Translation:* *Nashakaga kwibonera amafaranga menshi*

Not at all Extremely

Nta na rimwe Birenze/mu buryo bukabije

E. I was bored and it was a way to make it more entertaining. *Translation:* *Numvaga nabihiwe/narambiwe byari uburyo bwo kwishimisha*

Not at all Extremely

Nta na rimwe Birenze/mu buryo bukabije

F. I thought it was the aim of the experiment. *Translation:* *Natekerezaga ari yo ntego y’ubushakashatsi*

Not at all Extremely

Nta na rimwe Birenze/mu buryo bukabije

G. I was afraid to feel judged by the other persons present in the room. *Translation:* *Numvaga mfite ubwoba bw’ibyo abandi banu bari bari aho bari bumbonemo/bari bumvugeho*

Not at all Extremely

Nta na rimwe Birenze/mu buryo bukabije

H. My (family) education influenced my decisions. *Translation:* *Uburyo narezwe buri mu bwagize uruharemu myanzuro nagiye mfata*

Not at all Extremely

Nta na rimwe Birenze/mu buryo bukabije

I. The history of my country influenced my decisions. *Translation: Amateka y’igihugu cyanjye yagize uruhare mu myanzuro nagiye mfata*

Not at all Extremely

Nta na rimwe Birenze/mu buryo bukabije

J. For moral reasons. *Translation:* *Kubera indangagaciro zanjye*

Not at all Extremely

Nta na rimwe Birenze/mu buryo bukabije

K. ONLY FOR AGENTS FIRST. I was afraid to receive several electric shocks later when I would turn victim. *Translation:* *KU WABANJE MU GIKORWA CYO KOHEREZA MURI MUGNZI WE IMBARAGA Z’AMASHANYARAZI. Numvaga mfite ubwoba ko uwo noherezagamo amashanyarazi aza kunyishyura byinshi igihe nza kuba namusimbuye*

Not at all Extremely

Nta na rimwe kenshi bikabije

L. ONLY FOR VICTIMS FIRST. I had previously received many shocks, so it seemed fair. *Translation:* *KU WABANJE KOHEREZWAMO AMASHANYARAZI: Na we yabikoze inshuro nyinshi nagombaga kumwishyura*

Not at all Extremely

Nta na rimwe kenshi bikabije

6. If you disobeyed for other reasons please explain here. *Translation:* *Niba wanze gukora ibyo wasabwaga kubera indi mpamvu, gerageza kuyidusobanurira*

______________________________________________________________________________________________________________________________________________________________________________________________________________________________________________________________________________________________________________________________________________________________________________________

7. Please describe in a few word how did you feel during the experiment. *Translation: Gerageza kutubwira uko wiyumvaga igihe wamaze uri muri ibi bikorwa by’ubushakashatsi*

____________________________________________________________________________________________________________________________________________________________________________________________________________________________________________________________________________________________________________

***Supplementary Material S3***

#### Please answer as honestly as possible to the following sentence. *Translation:* *Ugerageze kuvugisha ukuri mu gusubiza ibi bibazo bikurikira*

1. If I had to rate how much my family has suffered during the genocide, what would be the score? *Translation:* *Iyaba nashobora gushyira ku gipimo uburyo umuryango wanjye wababaye mu gihe cya jenoside yakorewe abatutsi, nabiha ayahe manota?*

(Not suffered at all) **0 1 2 3 4 5 6 7 8 9 10** (Extremely)

Habe na gato birenze

1. How much do I think that the history of my family during the genocide influenced my current feelings, decisions, behaviors and actions? *Translation:* *Ni ku ruhe rugero numva amateka y’umuryango wanjye mu gihe cya genocide angiraho ingaruka ku byiyumviro byanjye magingo aya, gufata imyanzuro (ibyemezo), imyitwarire ndetse n’bikorwa?*

(Not at all) **0 1 2 3 4 5 6 7 8 9 10** (Extremely)

1. How frequently do I talk with my family of what happened to them during the genocide? *Translation:* *Ese ni ku ruhe rugero njya nganira n’abagize umuryango wanjye ku bijyanye n’ibyababayeho mu gihe cya genocide?*

(Never) **0 1 2 3 4 5 6 7 8 9 10** (All the time)

1. How many close family members of me has died during the genocide? Open question. *Translation:* *Ni abantu bangahe bo mu muryango wanjye wa hafi baba barapfuye bazize Genocide yakorewe abatutsi mu Rwanda?*

_____________________________________________________________________________________________________________________________________________________________________________________________________________________________________________________________________________________________________

1. How many relatives of me has suffered during the genocide? Open question. *Translation:* *Ni abantu bangahe bo mu muryango wanjye baba barahuye n’ibibazo cyangwa ihohoterwa iryo ari ryo ryose mu gihe cya genocide?*

____________________________________________________________________________________________________________________________________________________________________________________________________________________________________________________________________________________________________________

1. “Have you had the opportunity to participate to Peace Building and Reconciliation programs?” *Translation:* *Ese wigeze ugira amahirwe yo kwitabira cyangwa kugira uruhare mu gahunda zigamije kugarura ubumwe n’ubwiyunge?*Responses: YES - NO.
2. If YES, *“How frequently did you attend to peace building and reconciliation programs?”*. *Translation: Niba ari yego “ Ni ku ruhe rugero waba waritabiriye izo gahunda?”*

(Rarely) **0 1 2 3 4 5 6 7 8 9 10** (Very Frequently)

( Inshuro nkeya) ( Kenshi cyane)

1. *“How frequently do you listen to reconciliation radio programs?”*. *Translation:* *Ni ku ruhe rugero ujya wumva kuri radio ibiganiro bijyanye na gahunda z’ubumwe n’ubwiyunge*

(Rarely) **0 1 2 3 4 5 6 7 8 9 10** (Very Frequently)

( Inshuro nkeya) ( Kenshi cyane)

***Supplementary Material S4***

*Relational/personal identification with the experimenter (6 items)*

*Uko wihuza n’umushakashatsi*

- I identify with this experimenter. *Translation:* Niyumvamo uyu mushakashatsi

        strongly disagree       1      2      3      4      5      6      7       strongly agre

Simbyemera na gato Ndabyemera cyane

- I feel strong ties with this experimenter. *Translation: Numva mpujwe cyane n’uyu mushakashatsi*

        strongly disagree       1      2      3      4      5      6      7       strongly agree

Simbyemera na gato Ndabyemera cyane

- I am pleased with this experimenter. *Translation:* Nshimishijwe n’uyu mushakashatsi

        strongly disagree       1      2      3      4      5      6      7       strongly agree

Simbyemera na gato Ndabyemera cyane

- This experimenter endorses values that are important to me. *Translation:* Uyu mushakashatsi afite indagaciro z’ingenzi kuri njye

        strongly disagree       1      2      3      4      5      6      7       strongly agree

Simbyemera na gato Ndabyemera cyane

- This experimenter has values that are similar to my values. *Translation:* Uyu mushakashatsi afite indangagaciro zihuye n’izanjye

        strongly disagree       1      2      3      4      5      6      7       strongly agree

Simbyemera na gato Ndabyemera cyane

- This experimenter is a model for me to follow. *Translation:* Uyu mushakashatsi ni icyitegererezo kuri njyewe **nakurikiza**.

        strongly disagree       1      2      3      4      5      6      7       strongly agree

Simbyemera na gato Ndabyemera cyane

*Personal bond with the experimenter (4 items)*

- On a personal level, I can relate to this experimenter. *Translation:* Njye ku giti cyanjye nshobora kwihuza n’uyu mushakashatsi

        strongly disagree       1      2      3      4      5      6      7       strongly agree

Simbyemera na gato Ndabyemera cyane

- On a personal level, I feel a connection with this experimenter. *Translation:* Njye ku giti cyanjye numva numva hari ikimpuje n’uyu mushatashatsi

        strongly disagree       1      2      3      4      5      6      7       strongly agree

Simbyemera na gato Ndabyemera cyane

- On a personal level, I can bond with this experimenter. *Translation:* Ku giti cyanjye numva nahuza n’uyu mushakashatsi

        strongly disagree       1      2      3      4      5      6      7       strongly agree

Simbyemera na gato Ndabyemera cyane

- On a personal level, I feel close to this experimenter. *Translation:* Ku giti cyanjye numva mpuje cyane n’uyu mushakashatsi

        strongly disagree       1      2      3      4      5      6      7       strongly agree

Simbyemera na gato Ndabyemera cyane

*Experimenter charisma (5 items)* Urugwiro rw’umushakashatsi

- This experimenter is a charismatic leader. *Translation:* Uyu mushakashatsi ni umuyobozi ufite urugwiro

        strongly disagree       1      2      3      4      5      6      7       strongly agree

Simbyemera na gato Ndabyemera cyane

- This experimenter has a vision that spurs people on. *Translation:* Uyu mushakashatsi afite icyerekezo gitera abantu imbaraga

        strongly disagree       1      2      3      4      5      6      7       strongly agree

Simbyemera na gato Ndabyemera cyane

- This experimenter increases others’ optimism for the future. *Translation:* Uyu mushakashatsi yongerera abantu icyizere cy’ejo hazaza heza

        strongly disagree       1      2      3      4      5      6      7       strongly agree

Simbyemera na gato Ndabyemera cyane

- This experimenter has a special gift for seeing what is worthwhile for others to consider. *Translation:* Uyu mushakashatsi afite impano idasanzwe yo kumenya ibifitiye abandi agaciro byo kwitaho.

        strongly disagree       1      2      3      4      5      6      7       strongly agree

Simbyemera na gato Ndabyemera cyane

- This experimenter gives people a sense of overall purpose. *Translation:* Uyu mushakashatsi atera abantu kwiyumvamo intego nyamukuru.

        strongly disagree       1      2      3      4      5      6      7       strongly agree

Simbyemera na gato Ndabyemera cyane

***Supplementary Material S5.***

Each result was analyzed with both frequentist and Bayesian statistics (Dienes, 2011). Bayesian statistics assess the likelihood of the data under both the null and the alternative hypothesis. BF_10_ corresponds to the *p*(data|*H*_1_)/*p*(data|*H*_0_). Generally, a BF between 1/3 and 3 indicates that the data is similarly likely under the H_1_ and H_0_, and that the data does not adjudicate which is more likely. A BF_10_ below 1/3 or above 3 is interpreted as supporting H_0_ and H_1_, respectively. For instance, BF_10_=20 would mean that the data are 20 times more likely under H_1_ than H_0_  providing very strong support for H_1_, while BF_10_=.05 would mean that the data are 20 times more likely under H_0_ than H_1_ providing very strong support for H_0_ (Marsman & Wagenmakers, 2017). BF and p values were calculated using JASP (JASP Team, 2019) and the default priors implemented in JASP (Keysers et al., 2020). Default priors used in JASP depend on the statistical tests performed (for ANOVA, see Rouder et al., 2012; for t-tests, see Jeffreys, 1961; for correlations, see Jeffreys, 1961 and Ly et al., 2016). All analyses were two-tailed.

- Dienes, Z. (2011). Bayesian versus orthodox statistics: Which side are you on?. *Perspectives on Psychological Science*, *6*(3), 274-290
- Jeffreys, H. (1998). *The theory of probability*. OUP Oxford.
- Ly, A., Verhagen, J., & Wagenmakers, E. J. (2016). Harold Jeffreys’s default Bayes factor hypothesis tests: Explanation, extension, and application in psychology. *Journal of Mathematical Psychology*, *72*, 19-32
- Marsman, M., & Wagenmakers, E. J. (2017). Bayesian benefits with JASP. *European Journal of Developmental Psychology*, *14*(5), 545-555.
- Keysers, C., Gazzola, V., & Wagenmakers, E. J. (2020). Using Bayes factor hypothesis testing in neuroscience to establish evidence of absence. *Nature neuroscience*, *23*(7), 788-799.
- Rouder, J. N., Morey, R. D., Speckman, P. L., & Province, J. M. (2012). Default Bayes factors for ANOVA designs. *Journal of Mathematical Psychology*, *56*(5), 356-374.

***Supplementary Material S6.***

Paired comparisons between shock and no shock trials in the main experimental block confirmed that the P3, eLPP and lLPP were sensitive to the processing of the pain felt by the ‘victim’, with a higher amplitude for shock trial than for no shock trials (P3: t_(132)_=5.675, *p* < .001, Cohen’s d=.492, BF_10_=133635.51 - eLPP: t_(132)_=6.996, *p* < .001, Cohen’s d=.607, BF_10_=7.271e+7 - lLPP: t_(132)_=6.271, *p* < .001, Cohen’s d=.544, BF_10_=2.091e+6). This difference was in favor of H_0_ for the N1, P2 and N2 (all *p*s>.2, BFs_10_≤.192).

***Supplementary Material S7***

Paired comparisons between shock and no shock trials in the observation block confirmed that the P3, eLPP and lLPP were sensitive to the processing of the pain felt by the ‘victim’, with a higher amplitude for shock trial than for no shock trials (P3: t_(136)_=10.720, *p*<.001, Cohen’s d=.916, BF_10_=6.044e+16 - eLPP: t_(136)_=14.100, *p* < .001, Cohen’s d=1.205, BF_10_=1.582e+25 - lLPP: t_(136)_=12.735, *p*<.001, Cohen’s d=1.088, BF_10_=6.554e+21). This difference was slightly in favor of H_0_ for the N1, P2 and N2 (all *p*s>.4, BFs_10_≤.129).

***Supplementary Material S8***

Since our pain stimuli were not pre-recorded, the perceived intensity of the shock delivered to the ‘victim’ could vary between agents. In order to ensure that those who disobeyed the most were not those who observed a higher pain reaction on the victim’s hand, we performed a Pearson correlation between the subjective perception of the victim’s hand reported by the agent and prosocial disobedience. This correlation was in favor of H_0_ (*p*>.5, BF_10_=.133), thus ruling out the possibility that our positive correlation between prosocial disobedience and the neural processing of pain was stimulus-dependent.

***Supplementary Material S9***

*Self-reported personality traits and identification to the experimenter*

We then focused on possible differences in self-reported personality traits. One participant did not complete the Money Attitude Scale (MAS). Results indicated that the group tested in Rwanda scored higher on a scale measuring the degree to which people defer to authority (ASC scale: t_(142)_=7.219, *p*<.001, Cohen’s d=1.203, BF_10_=2.534e+8), moral foundations in decision-making (MFQ: t_(135.19)_=4.572, *p*<.001, Cohen’s d=0.762, BF_10_=1645.96), and on the importance of money (MAS: t_(141)_=9.562, *p*<.001, Cohen’s d=1.599, BF_10_=8.168e+14), see **Fig. S1A**. Full results for each subscale are displayed in **Table S1**.

**TABLE S1. Means, SDs and independent sample t-tests for each subscale between the group tested in Rwanda and the group tested in Belgium. Subscales in red bold differed statistically between the two groups.**

| Questionnaire | subscale | Group | mean | SD | t | df | *p* | Cohen‘s d | BF_10_ |
| --- | --- | --- | --- | --- | --- | --- | --- | --- | --- |
| Agression-Submission-Conventionalism scale  (ASC) | **Submission** | Rwanda | 8.16 | 1.38 | 8.055 | 142 | <.001 | 1.343 | 2.043e+10 |
|  |  | Belgium | 6.58 | 0.93 |  |  |  |  |  |
|  | **Aggression** | Rwanda | 10.05 | 1.04 | 4.995 | 142 | <.001 | 0.833 | 8641.29 |
|  |  | Belgium | 8.84 | 1.76 |  |  |  |  |  |
|  | Conventionalism | Rwanda | 8.00 | 1.15 | 0.331 | 142 | > .7 | 0.055 | 0.188 |
|  |  | Belgium | 7.93 | 1.35 |  |  |  |  |  |
| moral foundation questionnaire (MFQ) | Harm | Rwanda | 22.52 | 3.44 | -1.156 | 142 | > .2 | -0.193 | 0.330 |
|  |  | Belgium | 23.29 | 4.42 |  |  |  |  |  |
|  | **Firmness** | Rwanda | 21.33 | 3.84 | -4.223 | 142 | <.001 | -0.704 | 456.15 |
|  |  | Belgium | 24.04 | 3.85 |  |  |  |  |  |
|  | **Loyalty** | Rwanda | 19.83 | 3.71 | 5.590 | 142 | <.001 | 0.932 | 106542.04 |
|  |  | Belgium | 15.59 | 5.25 |  |  |  |  |  |
|  | **Authority** | Rwanda | 20.58 | 3.85 | 8.714 | 142 | <.001 | 1.452 | 7.436e+11 |
|  |  | Belgium | 14.33 | 4.70 |  |  |  |  |  |
|  | **Purity** | Rwanda | 21.19 | 3.53 | 5.795 | 142 | <.001 | 0.966 | 264677.05 |
|  |  | Belgium | 16.75 | 5.46 |  |  |  |  |  |
| Money Attitude Scale ( MAS) | **Power/prestige** | Rwanda | 22.27 | 10.76 | 8.791 | 141 | <.001 | 1.470 | 1.088e+12 |
|  |  | Belgium | 7.95 | 8.57 |  |  |  |  |  |
|  | **Retention/time** | Rwanda | 26.34 | 7.69 | 7.610 | 141 | <.001 | 1.273 | 1.875e+9 |
|  |  | Belgium | 15.64 | 9.06 |  |  |  |  |  |
|  | **Distrust** | Rwanda | 22.31 | 8.05 | 3.337 | 141 | =.001 | 0.558 | 26.04 |
|  |  | Belgium | 17.67 | 8.57 |  |  |  |  |  |
|  | **Anxiety** | Rwanda | 20.11 | 7.00 | 6.646 | 141 | <.001 | 1.112 | 1.418e+7 |
|  |  | Belgium | 12.78 | 6.13 |  |  |  |  |  |

Results also revealed higher identification to the experimenter reported by the group tested in Rwanda when compared to the group tested in Belgium (t_(142)_=7.449, *p*<.001, Cohen’s d=1.242, BF_10_=8.303e+8), see **Fig. S1B**. We then conducted multiple linear regressions with the subscales of each questionnaire – analyzed separately – to identify which subscales were the best predictors of prosocial disobedience. We centered the overall score of all subscales – considered as predictor variables in the linear regression - before building the model.


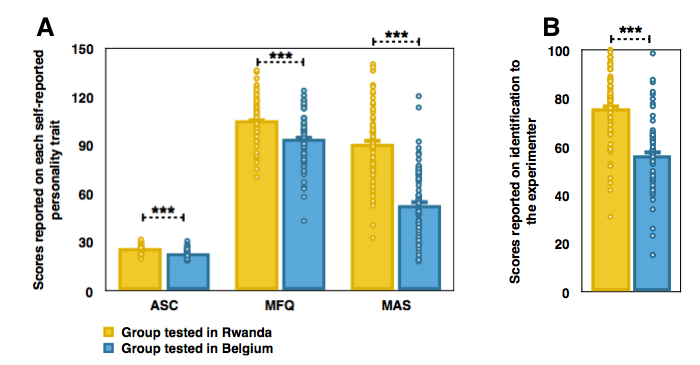


**Fig. S1.** A) Graphical representation of the comparison between the two groups in self-reported personality traits. ASC= Aggression-Submission-Conventionalism. MFQ=Moral Foundation Questionnaire. MAS=Money Attitude Scale. B) Graphical representation of the comparison between the two groups in the identification to the experimenter. All tests were two-tailed. *** represents a p≤.001 and a BF_10_≥3. Error bars represent standard errors. Colored dots represent individual data.

For the ASC questionnaire, only the submission subscale (e.g., “*People in positions of authority generally tell the truth*”) influenced prosocial disobedience (t_(143)_=-3.191, *p*=.002, Beta=-.265, BF_incl_=50.167). The lower participants scored on the submission scale, the higher was their prosocial disobedience. The aggression and conventionalism subscales were respectively inconclusive (*p* > .1, BF_incl_=.591) and in favor of H_0_ (*p* >.5, BF_incl_=.282). All VIFs were below 2.

For the moral foundation questionnaire (MFQ), the harm subscale (e.g., “*Whether or not someone suffered emotionally*” is important to decide is something is right or wrong) was the best predictor of prosocial disobedience (t_(143)_=2.460, *p*=.015, Beta=.236, BF_incl_=6.421). The more participants value protecting vulnerable individuals from harm, the more they disobeyed in a prosocial way. The authority subscale was in favor of H_1_ with the Bayesian approach (BF_incl_=4.292) but not significant with the frequentist approach (t_(143)_=-1.546, *p*=.12, Beta=-.190). The lower participants value authority (e.g., “*Whether or not someone showed a lack of respect for authority*” is important to decide is something is right or wrong), the more they disobeyed prosocialy. Other subscales (i.e., firmness, loyalty and purity) were inconclusive (all *p*s > .1, all BFs_incl_ ≤1.098 & ≥.444). All VIFs were below 3.

For the money attitude scale (MAS), we excluded from the analysis the variant of the task which did not include a monetary reward (i.e. Do not decide + No monetary gain). Participants were indeed informed that they would be paid for this experiment no matter their decision during the task in this variant and did not receive money in exchange for the shocks sent. The power subscale (e.g., “*I use money to influence other people to do things for me*”) was in favor of H_1_ with the Bayesian approach (BF_incl_=3.497) but not significant with the frequentist approach (t_(95)_=-1.555, *p*=.12, Beta=-.234). All VIFs were below 3.

For the identification with the experimenter, we observed with the frequentist approach that the personal bond subscale (e.g. “*On a personal level, I can relate to this experimenter*”) was the best predictor of prosocial disobedience (t_(143)_=-2.183, *p*=.031, Beta=-.278). With the Bayesian approach, there was only an anecdotal evidence (BF_incl_=2.648). The more participant felt a personal bond with the experiment, the less they disobeyed. Other subscales (i.e., personal identification, charisma) provided anecdotal evidences for H_0_ (all *p*s > .1, all BFs_incl_ ≤.385 & ≥.759). All VIFs were below 3.

***Supplementary Material S10***

*Family suffering during the genocide*

We then compared the reported family suffering during the 1994 genocide in order to ensure that the two groups did not experience differently the family trauma due to the genocide. One participant did not answer this question. Results supported no statistical differences between Rwandese tested in Rwanda and Rwandese tested in Belgium (*p* > .4, BF_10_=.232), with an average score of 8.29 (CI_95_=7.9-8.6) on a scale ranging from ‘0’ (not suffered at all) to ‘10’ (suffered extremely), denoting the importance of the suffering of their family during the 1994 genocide. The group tested in Rwanda nonetheless reported talking more frequently about the 1994 genocide with their family (5.8, CI_95_=5.2-6.5) than the group tested in Belgium (4.5, CI_95_=3.8-5.1, t_(141)_=2.428, *p* = .016, Cohen’s d=.406). The Bayesian version of the same analysis reported an anecdotal evidence for H_1_ (BF_10_=2.586). The two groups did not differ regarding how much they think that their behaviors are influenced by their family history during the genocide (*p* > .4, BF_10_=.226). A multiple linear regression with prosocial disobedience as the dependent variable and family suffering during the 1994 genocide, how much participants think that their family history during the genocide influenced their behaviors, and how frequently participants talk about those events with their family as the independent variables showed that only family suffering during the genocide was a predictor variable of prosocial disobedience (t_(142)_=2.554, *p*=.012, Beta=.233, BF_incl_=4.401). The correlation indicated that participants who disobeyed the most frequently were also those who indicated a higher family suffering, see **Fig. 2C**. How frequently participants talked about those events with their family (*p* >.7, BF_incl_=.260) and how much participants think that their family history during the genocide influence their behaviors (*p* > .8, BF_10_=.255) did not influence prosocial disobedience. The variance inflation factors (VIF) were of 1.2, thus confirming the absence of collinearity in our model. We further ran a Pearson correlation between the pain response and the reported family suffering during the genocide. This correlation showed evidence for a positive association between the reported family suffering during the genocide and the amplitude of the neural response to the pain of others (r=.247, *p* = .004, BF_10_=6.938), see **Fig. 2D**.

***Supplementary Material S11***

**Reasons for prosocial disobedience.** All participants who reported that they voluntarily disobeyed the orders of the experimenter were presented a list of 10 reasons that they had to rate from “Not at all” to “Extremely” (see ***Supplementary Material S2***). We performed Pearson correlations between the percentage of prosocial disobedience and the different reasons reported. We applied Bonferroni corrections to our correlations (α/10=.05/10=.005) with the frequentist approach. Both frequentist and Bayesian data indicated that the percentage of prosocial disobedience positively correlated with moral reasons (r=.495, *p*<.001, BF_10_=939.06): The higher the percentage of prosocial disobedience was, the higher the reason associated with moral values was. Results also indicated that the more agents felt bad for the ‘victim’, the higher the prosocial disobedience was (r=.445, *p*<.001, BF_10_=146.6). We further observed that the less participants were interested in making money, the more they refused immoral orders (r=-.361, *p*=.007, BF_10_=5.630). To what extent participants though that disobedience was the aim of the experiment was in favor of H0 (*p*>.2, BF_10_=.281). Other correlations supported a lack of sensitivity (all *p*s>.016, all BFs_10_≥.369&≤2.586).

Independent sample t-tests were also performed to observe the weight of each reason for prosocial disobedience between the group tested in Belgium and the group tested in Rwanda. Results indicated that Rwandese tested in Belgium reported a higher weight of moral reasons (t_(64)_=-3.061, *p*=.003, Cohen’s d=-.832, BF_10_=11.677) and reasons associated with the (too high) number of shock to deliver to the ‘victim’ (t_(64)_=-2.748, *p*=.008, Cohen’s d=-.747, BF_10_=5.764) than the group tested in Rwanda, see **Fig. S2**. The group tested in Rwanda reported that being afraid to be judged by the other persons present in the room had more weight to explain prosocial disobedience than the group tested in Belgium (t_(64)_=4.930, *p*<.001, Cohen’s d=1.340, BF_10_=2522.28). Other comparisons were in favor of H_0_ or indicated a lack of sensitivity (all *p*s>.02, all BFs_10_≥.276&≤2.806).

**Fig. S2.** Graphical representation of the weight for each reason given by both groups to justify prosocial disobedience. All tests were two tailed. * represents a *p*≤.05 and a BF_10_≥3. Error bars represent standard errors.

**Reasons for obeying.** If participants reported that they did not voluntarily disobey the orders of the experimenter, they were asked in an open question to explain their decision to comply with those orders. Three categories were extracted from the reasons provided: (1) ‘For science’ reasons; participants reported that they obeyed to allow reliable data acquisition (e.g., “*Pour ne pas fausser l’étude*” – English translation: “*To avoid biasing the stud*y”); (2) ‘For respect of authority’ reasons; participants reported that they had to follow the orders of the authoritative figure (e.g., “*It is because I took it as my duties to follow the instructions of my guider*”), and (3) ‘for lack of side-effects’ reasons; participants reported that since the shocks delivered were calibrated on one’s own pain threshold, obeying orders to shock were not tricky (e.g., “*I was thinking that the threshold was not too high*”). An independent, naive judge classified the response of participants in one or several of those three established categories. Results indicated that participants reported that their reasons for obeying orders were mostly due to their willingness to provide good data (i.e. “for science”, reported 26/39 times) and because they respect hierarchy (i.e., “For respect of authority”, reported 22/39 times). 14/39 mentioned the lack of side effects.

**Individual responses of participants for reasons for obeying orders from the experimenter** (translated in English by the authors if the original answer was in Kinyarwanda or in French).

| Participant | Reasons for NOT disobeying the orders of the experimenter |
| --- | --- |
| P1 | I didn't disobey intentionally because I respect each and every person who is guiding me |
| P2 | I was very vigilant |
| P3 | I didn't disobey in order to ensure efficient results of the research and being truthful |
| P4 | I felt I must obey all the instructions because I wanted the experiment to the fruitful, I never wanted to ruin anything in the process |
| P5 | The experiment was clearly explained to me, the experiment was interesting, I thought that any sort of disobedience would lead to losing the compensation fees |
| P6 | To facilitate the experimenters with their goals |
| P8 | I have tried my best to follow order because my victim was not responding with signs of pain, so I thought it was not painful to him |
| P9 | My reason for not disobeying is that before the experiment I was given instructions to follow. So, I followed the given instruction of pressing the button (shock/no sock) I was told to press |
| P10 | I have been explained about the procedures about how experiment should be done so I had to pay attention from experimenter’s instructions as was requested before starting the experiments |
| P11 | I was interested in your research so that you'll get accurate information |
| P12 | It is to help the researchers to accomplish their study. It was fun to see how electrical charges can stimulate muscle cells and nerve cells |
| P13 | I followed the orders so that the researchers may achieve their goals of the experiment and imagined that it is not so painful to the victim |
| P15 | I followed the order because I have well understood the purpose of the experiment |
| P16 | Because I know I'm the one responsible to send a shock |
| P18 | I thought that the experiment will get results because of me not obeying the instructions |
| P19 | Because it is better to always obey order given by your supervisor in the way experiment is carried out |
| P20 | The reason was because I was the one who chose the extent of pain which was used during the experiment, which was quite simple and I was told there is no side effect at all |
| P21 | As I was told to follow instructions, I did follow them |
| P22 | Because they have given the explanations before and am doing it as volunteer so I have to obey the instructions |
| P23 | I didn't commit errors willingly because I wish to be a part in medical field development and I had no idea on how the data collected would be interpreted so I didn't want to bias the data |
| P24 | I disobeyed only once due to lack of attention |
| P25 | Due to the fact that I voluntarily participate in this experiment and after starting the experiment I signed consent means all terms and conditions belong to this experiment must be considered I obey to follow rule and regulation regarding to this experiment because is sign consent a voluntarily participate |
| P27 | I have followed all commands given to me that why I have not disobeyed to send signals or to be a victim |
| P28 | Because we accept it before starting experiment |
| P29 | I thought that disobeying the instructions intentionally could give the negative (or false positives) on the experiment results |
| P30 | I think I was to obey the command as a part of research |
| P32 | I came to participate in this experiment so as I can learn more that will help me in the future life may be or that I can also explain to others how it works. So I had to follow the orders of experiments to be more experienced |
| P33 | They have already explained to us before experiment, they mentioned to us that no harm and usually is like research project because I know no research which is meaningless, all researchers aim positive ambitions to change the world and living |
| P34 | Disobeyed for the pain score |
| P36 | I was following the instructions of you only (no other reason) |
| P37 | It is because I took it as my duties to follow the instructions of my guider |
| P38 | I was following instructions so that I participate to having good results at the end of this research |
| P39 | I suggested that there is no much pain and I wanted to estimate my scale according to shock and no shock. But there is so much time for practicing this incidentally sometimes it was unwilling to click on shock button |
| P41 | I didn't disobey because it was among the request and order I was given. I think that to disobey can disturb/ can lead to poor result of research |
| P42 | I heard/followed the instructor carefully in order to not skip any question unanswered/ unfilled space, so I tried to stay focused to the instruction and what I was requested to do. |
| P43 | For the success of the study |
| P45 | I followed orders in order not to bias the intended observation |
| P46 | Because the orders were explained well to me and there was no risk to my colleague |
| P48 | Respect for command is very important because I think that the commander ( the one who was commanding me) instruct me for a certain reason so choose to obey |
| P49 | Because I was supposed to accomplish my duties (to play my role) |
| P50 | For the research to be successful |
| P51 | Because I want to make a good research finding if I promise thing to do I do it correctly, I used to obey all commands if no harmful to my colleague. I want to make things perfectly |
| P53 | I did it to respect the order of experiment as I have signed the consent form |
| P54 | It's just because of sacrificing myself for proving and improving quality of research in all over the world. Another reason is that 've been explained well the steps and rules of the research |
| P55 | I did everything that I was supposed to do because it was my responsibility and to not cause any bias in results that will come from this research |
| P56 | I did not disobey because the victim does not react like an individual who is experiencing pain means that he is reacting as normal |
| P59 | I followed orders throughout the experience, to make the study valid and avoid making confusion as I wanted to participate, I did not have a reason for creating a bias as I was well explained the task to do and to follow so it was not complicated |
| P60 | I didn't hesitate to apply any command because even though I had been fearing because thinking about the pain I had been given in advance that the pain is not severe to be tolerated |
| P63 | No one forced me to participate in the research, so I had to follow instructions |
| P64 | I followed the instruction given as I knew that they will be no harm to the victim |
| P65 | I was thinking that the threshold was not too high |
| P66 | The reason for following order is that as I got instructions before experience, the instructions was about to press shock or not as followed from the person who is giving instructions, so I followed instructions as was given in between experiment |
| P67 | I just respect the instructions for making the research successful |
| P68 | I did that to avoid any bias in this experiment |
| P71 | Because it would interfere with the research. Research results could be inaccurate |
| P72 | I was following the orders through the experiment because I was thinking there is not much pain to the victim |
| P98 | - For the sake of the experiment |
| P99 | Because I had the impression that it was not painful for her |
| P100 | I accepted to participate in this study, so I decided to play the game |
| P102 | I prefer to do what I am asked to do rather than disobeying |
| P106 | It was not that painful as victim, so I imagined that it was not that painful for the other participant |
| P107 | I followed the orders because I knew it was not that painful and because it was the goal of the experiment. I executed the orders without even thinking about them and it was not a problem for me to send those shocks. |
| P110 | Because of the experiment – I felt it was an obligation to follow the orders of the experimenter |
| P111 | Since we were participating in an experiment, I have done what I was told to do and I don’t think I really hurt the victim since the pain was calibrated |
| P118 | I thought it was better to follow orders. I felt that I had to do what I was told and I thought it was good for the experiment. |
| P120 | I could not see a single reason for disobeying |
| P123 | I knew that even if I was following orders, there won’t be any severe consequences |
| P124 | Conditioning to follow orders during an experiment to please the experimenter |
| P125 | I felt the need to follow the orders I was receiving |
| P126 | For the sake of the experiment |
| P131 | I did not feel that I had the choice. I wanted to disobey but the shocks did not appear to be highly severe. I would have disobeyed if the shocks would have been severe. |
| P132 | To respect the instructions of the experiment |
| P134 | So the experiment could be conclusive |
| P136 | Because it was the instructions and I wanted the experiment to be successfully conducted. |
| P140 | Since it was an experiment, I did not disobey even if I wanted to. But now I regret not to have disobey. |
| P141 | It did not appear to be painful |
| P142 | - Respect of the experiment - Respect of the rules - Revenge (because I was victim first) - No visual information from the victim - No severe consequences - Respect for the experimenter |
| P144 | I followed the orders because I was given a mission. Since I was victim first, I had the feeling that the shocks were not unbearable |
| P145 | I have no reasons to send a shock a shock to the other person |
| P148 | Because I don’t take any risks if I don’t know the consequences of disobeying |
| P163 | Because I had to follow the rules and not hurt more the victim |

***Supplementary Material S12.***

Antisocial disobedience happened in average on 14.10 % (SD=3.03) for participants who explicitly reported that they voluntarily disobeyed during the task. Exploratory correlations were conducted between the antisocial disobedience rate and the different self-reported questionnaires, as well as how responsible, bad and sorry participants felt during the task on all participants (N=144). Uncorrected results are presented in **Table S2**. Higher antisocial disobedience rates indicate that participants more frequently refused the order of the experimenter not to send a shock. Uncorrected significant results are displayed in green.

**Table S2. Uncorrected correlation results between antisocial disobedience and self-reported questionnaires, how responsible, sorry and bad participants felt, how they estimated the victim’s pain and family suffering.**

|  | **Responsibility ratings** | **How bad?** | **How sorry?** | **Estimated pain of the victim** | **ASC submission** | **ASC aggression** | **ASC convention** |
| --- | --- | --- | --- | --- | --- | --- | --- |
| **Antisocial**  **disobedience** | r=-.091  *p* =.279 | r=-.188  *p* =.024 | r=-.209  *p* =.012 | r=-.210  *p* =.012 | r=.068  *p* =.416 | r=.052  *p* =.538 | r=.066  *p* =.434 |
|  | **DT Machiavellism** | **DT Narcissism** | **DT Psychopathy** | **MFQ Harm** | **MFQ Fairness** | **MFQ Loyalty** | **MFQ Authority** |
| **Antisocial**  **disobedience** | r=.206  *p* =.013 | r=.168  *p* =.045 | r=.122  *p* =.146 | r=-.255  *p* =.007 | r=-.291  *p* =.000 | r=.035  *p* =.678 | r=.011  *p* =.898 |
|  | **MFQ**  **Purity** | **IRI Empathic concern** | **IRI**  **Perspective-Taking** | **IRI**  **Personal distress** | **IRI**  **Fantasy** | **MAS power** | **MAS Retention** |
| **Antisocial**  **disobedience** | r=.022  *p* =.795 | r=-.291  *p* =.000 | r=-.146  *p* =.081 | r=-.110  *p* =.188 | r=-.057  *p* =.495 | r=.158  *p* =.059 | r=.136  *p* =.106 |
|  | **MAS**  **distrust** | **MAS**  **Anxiety** | **Experimenter**  **Personal Identification** | **Experimenter**  **Personal bond** | **Experimenter Charisma** | **Family suffering** |  |
| **Antisocial**  **disobedience** | r=.007  *p* =.931 | r=-.079  *p* =.347 | r=.103  *p* =.221 | r=.126  *p* =.134 | r=.078  *p* =.353 | r=-.153  *p* =.067 |  |

**Table S3** displays exploratory correlations between antisocial disobedience and reasons provided by participants who voluntarily disobeyed (N=66).

**Table S3.** Uncorrected correlation results between antisocial disobedience and reasons for disobedience

|  | **I felt bad** | **There were too many shocks** | **By contradiction** | **I wanted to make more money** | **I felt bored** |
| --- | --- | --- | --- | --- | --- |
| **Antisocial**  **disobedience** | r=-.698  *p* =.000 | r=-.359  *p* =.003 | r=-.168  *p* =.178 | r=.443  *p* =.001 | r=.179  *p* =.150 |
|  | **I thought it was the aim of the experiment** | **I did not want to be judged by others** | **My family education influenced my decision** | **The history of my country influenced my decision** | **For moral reasons** |
| **Antisocial**  **disobedience** | r=.113  *p* =.368 | r=.157  *p* =.209 | r=-.212  *p* =.090 | r=-.194  *p* =.119 | r=-.590  *p* =.000 |
